# Rfx6 Maintains the Functional Identity of Adult Pancreatic β Cells

**DOI:** 10.1016/j.celrep.2014.11.033

**Published:** 2014-12-11

**Authors:** Julie Piccand, Perrine Strasser, David J. Hodson, Aline Meunier, Tao Ye, Céline Keime, Marie-Christine Birling, Guy A. Rutter, Gérard Gradwohl

**Affiliations:** 1 Institut de Génétique et de Biologie Moléculaire et Cellulaire, Institut National de la Santé et de la Recherche Médicale U964, Centre National de Recherche Scientifique UMR7104, Université de Strasbourg, Illkirch 67404, France; 2 Section of Cell Biology, Division of Diabetes, Endocrinology and Metabolism, Department of Medicine, Imperial College London, Hammersmith Hospital, du Cane Road, London W12 0NN, UK; 3 Institut Clinique de la Souris-ICS-MCI, PHENOMIN, Illkirch 67404, France

## Abstract

Increasing evidence suggests that loss of β cell characteristics may cause insulin secretory deficiency in diabetes, but the underlying mechanisms remain unclear. Here, we show that Rfx6, whose mutation leads to neonatal diabetes in humans, is essential to maintain key features of functionally mature β cells in mice. Rfx6 loss in adult β cells leads to glucose intolerance, impaired β cell glucose sensing, and defective insulin secretion. This is associated with reduced expression of core components of the insulin secretion pathway, including glucokinase, the Abcc8/SUR1 subunit of K_ATP_ channels and voltage-gated Ca^2+^ channels, which are direct targets of Rfx6. Moreover, Rfx6 contributes to the silencing of the vast majority of “disallowed” genes, a group usually specifically repressed in adult β cells, and thus to the maintenance of β cell maturity. These findings raise the possibility that changes in Rfx6 expression or activity may contribute to β cell failure in humans.

## Highlights

• Inactivation of Rfx6 in adult β cells leads to impaired insulin secretion
• Glucose-induced Ca^2+^ influx is reduced in β cells lacking Rfx6
• Rfx6 activates components of the insulin secretion pathway
• Rfx6 maintains β cell maturity by repressing “disallowed” genes

## Graphical Abstract

**Figure:**
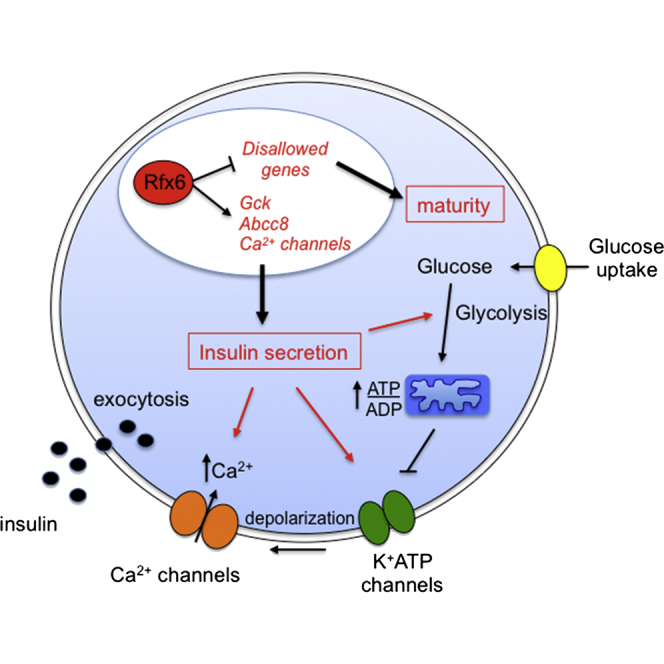

## Teaser

Rfx6 transcription factor is critical for the development of pancreatic β cells, and Rfx6 mutations cause neonatal diabetes in humans. Using adult β cell deletion of Rfx6, Piccand et al. show that Rfx6 controls glucose homeostasis and maintains the differentiated state by activation of the insulin secretory pathway and repression of disallowed genes, a group of genes selectively repressed in mature β cells.

## Introduction

The mammalian pancreas comprises an exocrine compartment, secreting digestive enzymes into the intestine, and an endocrine compartment, secreting hormones in the bloodstream. Pancreatic endocrine cells are grouped in small clusters of cells, the islets of Langerhans, containing different cell types secreting distinct hormones. Islet cells include β cells, which secrete insulin, the hormone stimulating glucose uptake in peripheral tissues. Briefly, glucose enters β cells by facilitated diffusion and, after phosphorylation by glucokinase (Iynedjian, 1993), is metabolized by aerobic glycolysis (Sekine et al., 1994), producing metabolic signals such as a rise in ATP/ADP concentration (Tarasov et al., 2012). The latter in turn closes ATP-sensitive K^+^ channels, causing membrane depolarization and the subsequent opening of voltage-gated Ca^2+^ channels (Yang and Berggren, 2006). Ca^2+^ influx then stimulates the exocytosis of insulin granules (Rutter, 2004).

Diabetes is a chronic metabolic disease characterized by hyperglycemia due to defective insulin secretion, insulin action, or both. β cells are lacking in type 1 diabetes, while in type 2 diabetic patients, β cells cannot compensate for the increased insulin demand due to their reduced capacity to secrete insulin in response to high blood glucose. Alterations in both β cell mass (Butler et al., 2003; Marselli et al., 2013; Rahier et al., 2008) and function (Rosengren et al., 2012) are likely to contribute to the overall secretory deficiency observed in type 2 diabetes (Rutter, 2014). Recently, it has been proposed that β cell dysfunction in type 2 diabetes might also result from a mechanism of dedifferentiation, which would compromise β cell function (Talchai et al., 2012) and contribute to the development of the disease together with cell death and decreased β cell mass. This hypothesis, which builds on earlier findings (Jonas et al., 1999), has been based on the observation that ablation of FoxO1 transcription factor in adult β cells in mice caused hyperglycemia with a concomitant reversion of β cells to a progenitor- or α-like state. Along the same lines, additional loss-of-function studies in adult β cells revealed that NeuroD1 (Gu et al., 2010), Nkx6.1 (Taylor et al., 2013), or Pdx1 (Gao et al., 2014) transcription factors are important to maintain the maturity and differentiated state as well as the insulin-secretive function of β cells. Thus, it appears that the loss of key β cell transcription factors results in the loss of both β cell identity and function.

Rfx6 is a winged-helix transcription factor that has been shown to be essential for islet cell development in zebrafish (Soyer et al., 2010), *Xenopus* (Pearl et al., 2011), mice (Smith et al., 2010), and humans (Concepcion et al., 2014; Pearl et al., 2011; Smith et al., 2010; Spiegel et al., 2011). *Rfx6* null mice lack all endocrine cells (excepting PP cells), including β cells, and die shortly after birth. It was thus concluded that Rfx6 is necessary for insulin production during embryogenesis (Smith et al., 2010). In humans, mutations in *RFX6* have been reported to be the cause of the Mitchell-Riley syndrome, an autosomal-recessive syndrome of neonatal diabetes and small bowel atresia, often associated with intestinal malabsorption (Concepcion et al., 2014; Smith et al., 2010; Spiegel et al., 2011). Clusters of chromogranin A-positive hormone-negative cells have been reported in the pancreas of several patients, suggesting a critical role for RFX6 in the formation of islet and β cells in humans. The complex spatiotemporal expression pattern of Rfx6 in mice, namely its broad expression very early in the gut and pancreas endoderm and then its restriction to developing endocrine cells in the embryo and its maintenance in adult islets, including β cells (Smith et al., 2010; Soyer et al., 2010), suggests multiple functions at different stages and in different organs. Thus, the phenotype of *Rfx6* null mice might result from multiple deficiencies during development. Importantly, the postnatal lethality of *Rfx6*^−/−^ pups precluded the study of Rfx6 function in adult β cells. Therefore, to decipher the multiple functions of Rfx6, we generated a floxed allele. Conditional inactivation of *Rfx6* in developing endocrine cells phenocopies the null phenotype, demonstrating that Rfx6 controls islet development downstream of the proendocrine transcription factor Ngn3. Conditional inactivation of *Rfx6* in adult β cells led to insulin secretion deficiency and glucose intolerance, although insulin was still produced. Removal of *Rfx6* perturbs key molecular traits of functional β cells with the reduction in expression of glucokinase, the ATP-sensitive K^+^ channel *Abcc8/SUR1*, as well as eight voltage-gated Ca^2+^ channel genes, some of which are direct targets of Rfx6. Collectively, lowered levels of these core components of the insulin secretion pathway seem likely to underlie defective insulin secretion. Unexpectedly, we found that inactivation of *Rfx6* in adult β cells caused the re-expression of the “disallowed” genes (Pullen et al., 2010; Quintens et al., 2008; Thorrez et al., 2011), a set of genes expressed normally in most (if not all) mammalian tissue but selectively and highly repressed in mouse mature β cells. This finding reveals a common repression mechanism of disallowed gene expression in β cells involving a unique transcription factor. Taken together, our data show that *Rfx6* inactivation in β cells causes decreased expression of β cell-specific genes combined with the upregulation of disallowed genes, a feature of immature β cells, demonstrating that Rfx6 can act as an activator or repressor of transcription. Thus, the present study demonstrates that Rfx6 is essential for the maintenance of the differentiated state and functional identity of adult β cells. Therefore, Rfx6 might be an interesting target should “β cell-identity drugs” be developed in the future as a novel therapeutic strategy in type 2 diabetes.

## Results

### Rfx6 Is Essential for β Cell Differentiation Downstream of the Proendocrine Transcription Factor Ngn3

**Figure 1:**
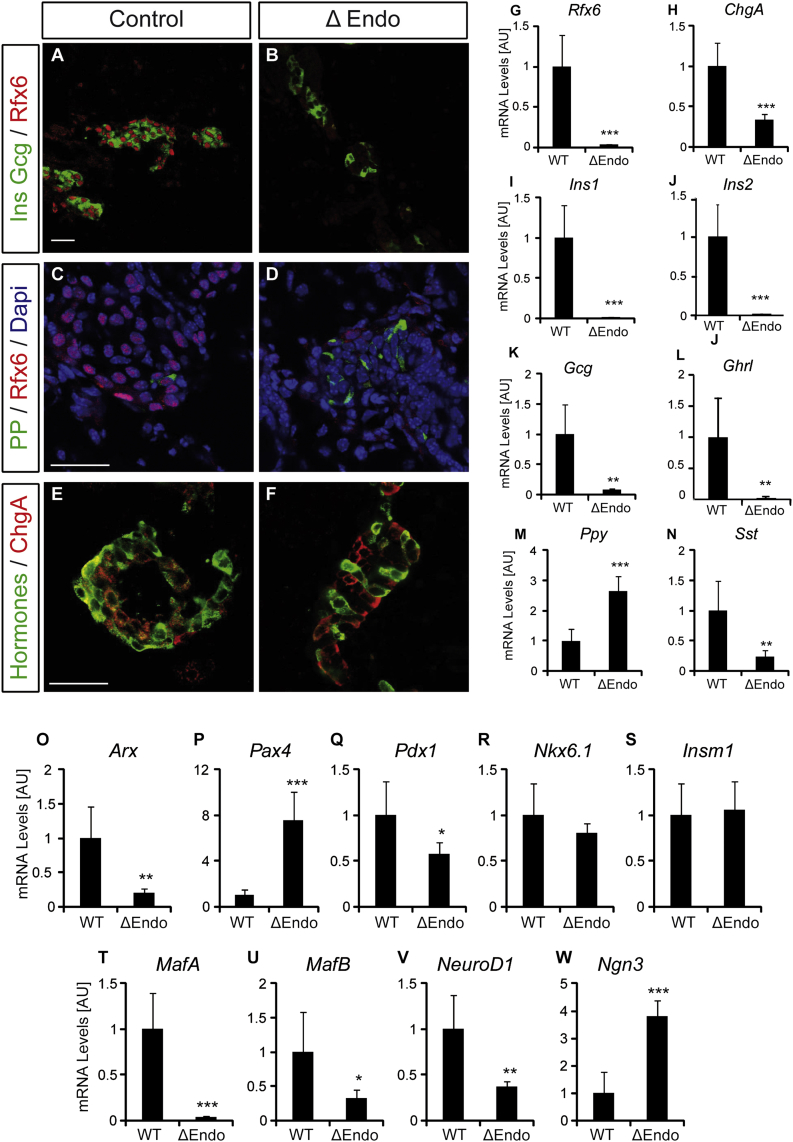
Deletion of *Rfx6* Downstream of *Ngn3* Results in the Loss of Insulin-, Glucagon-, Somatostatin-, and Ghrelin-Producing Islet Cells in Newborn Mice (A–F) Immunofluorescence experiments on pancreata from controls and Rfx6^ΔEndo^ pups at postnatal day 0 (P0). Staining for insulin and glucagon (green) and Rfx6 (red) showing efficient deletion of Rfx6 and strong reduction of insulin- and glucagon-expressing cells in Rfx6^ΔEndo^ mutants (A and B). (B) and (F) show very rare sections where hormone-positive cells were found. Staining for PP (green) and Rfx6 (red) revealed that PP is not dependent on Rfx6 (C and D). Staining for hormones (insulin, glucagon, PP, somatostatin) in green and the panendocrine marker chromogranin A in red show that chromogranin A-positive endocrine cells, which do not express any of the islet hormones, are found in the pancreas of Rfx6^ΔEndo^ pups (E and F). (G–W) qRT-PCR experiments for *Rfx6*, hormones, and transcription factors controlling islet cell development in Rfx6^ΔEndo^ pups and wild-type controls at P0. Scale bars, 50 μM. Data are presented as mean ± SD on n = 4 samples; ^∗∗∗^p < 0.001, ^∗∗^p < 0.01, ^∗^p < 0.05.

To decipher the different roles of Rfx6, we generated a conditional knockout mouse by flanking exon 3, which encodes a part of the DNA binding domain, by two *loxP* sites, generating a null allele (Figure S1). To determine the role of Rfx6 specifically in the endocrine lineage and circumvent any effect of a possible endodermal function, we first generated Rfx6^ΔEndo^ mutant animals by crossing *Rfx6*^fl/fl^ mice with Ngn3-Cre mice (Yoshida et al., 2004). *Rfx6* is efficiently deleted in Rfx6^ΔEndo^ pancreas (Figure 1G), and like the full-body knockout, Rfx6^ΔEndo^ pups are diabetic and die between 2 and 3 days after birth (not shown). *Ins1*, *Ins2*, *Gcg*, *Sst*, and *Ghr* mRNAs are strongly decreased, while the amount of *Ppy* transcripts is increased (Figures 1I–1N). Accordingly, the differentiation of hormone-expressing cells was strongly impaired (except for PP), as insulin- glucagon- or somatostatin-expressing cells are hardly found (Figures 1A–1D). However, although the overall amount of *ChgA* mRNA is decreased (Figure 1H), an endocrine program has been implemented since chromogranin A-positive but hormone-negative cells are found (Figures 1E and 1F). We further characterized the role of Rfx6 downstream of Ngn3 by exploring the expression levels of several important transcription factors controlling islet cell fate and maturation. Interestingly, *Ngn3* transcripts were elevated 4-fold in the pancreas of Rfx6^ΔEndo^ mutants at birth, suggesting that after being induced by Ngn3 in endocrine progenitors, Rfx6 would in turn repress *Ngn3* in developing islet cells or that the number of Ngn3 cells increases (Figure 1W). Cell quantification did not validate the later hypothesis (not shown). *Arx* transcripts are low, while those encoding *Pax4* are increased (Figures 1O and 1P). Thus, as proposed for the full knockout at embryonic stages (Smith et al., 2010), Rfx6 might control islet subtype specification by repressing the β fate while promoting α destiny in Rfx6^ΔEndo^ mice (see Discussion). This increase in *Pax4* was, however, not sufficient to induce β cell development, because the expression of key regulators of insulin transcription (*Pdx1*, *MafA*, *NeuroD1*) as well as *Ins1* was reduced (Figures 1Q, 1T, and 1V). Together, and as reported in *Rfx6* null embryos (Smith et al., 2010), Rfx6^ΔEndo^ newborns almost entirely lack insulin-positive cells. These findings demonstrate that it is Rfx6 function, downstream of Ngn3, that is essential for β cell generation and that impaired β cell development does not result from defective endoderm.

### Rfx6 Is Not Absolutely Necessary for Insulin Production in the Adult Mouse Pancreas

**Figure 2:**
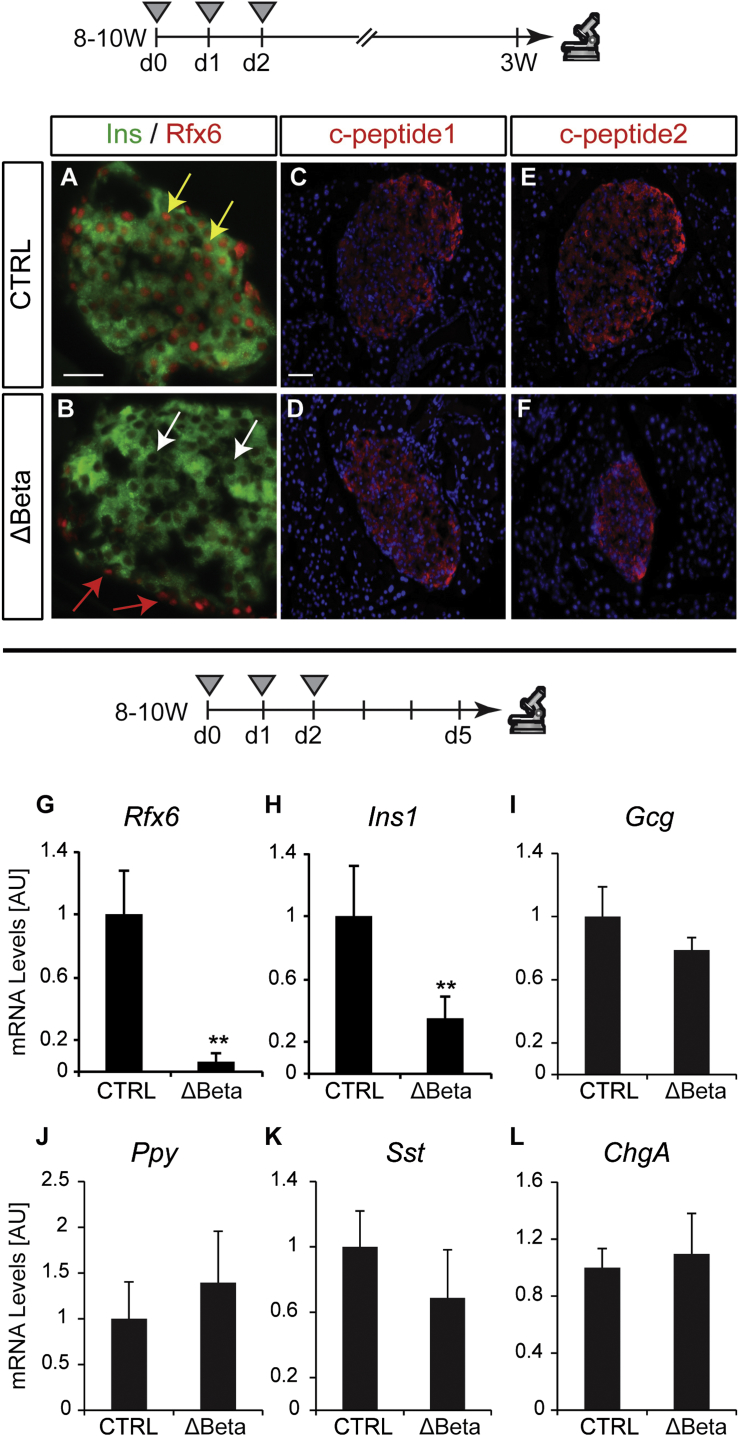
Insulin Is Produced in Adult β Cells Lacking Rfx6 (A–F) Immunofluorescence staining on controls and Rfx6^ΔBeta^ (Rfx6^fl/fl^; Ins1-CreERT^2^) adult mice, 3 weeks after the first day of tamoxifen injections (8- to 10-week-old mice were injected once a day during 3 consecutive days). Staining for insulin (green, A and B) and Rfx6 (red, A and B) reveal insulin expression despite efficient deletion of Rfx6 in β cells of Rfx6^ΔBeta^ mice (white arrows in B). Staining for c-peptide1 (red, C and D) and c-peptide2 (red, E and F) supports efficient insulin synthesis. (G–L) qRT-PCR experiments on islets purified from controls and Rfx6^ΔBeta^ adult (8- to 10-week-old) mice 5 days after tamoxifen injections revealing rapid and specific deletion of *Rfx6* (G) in β cells and decreased *Ins1* transcription (H), while the expression of *Gcg*, *Ppy Sst* and *ChgA* is unaltered. Grey triangles indicate the days of tamoxifen injections. Yellow and red arrows point to examples of β cells expressing Rfx6 in controls and insulin-negative/Rfx6-positive cells in Rfx6^ΔBeta^ mice, respectively. Scale bars, 50 μM. Data are presented as mean ± SD on n = 4 samples; ^∗∗∗^p < 0.001, ^∗∗^p < 0.01, ^∗^p < 0.05.

Rfx6 expression is maintained in adult islets, including β cells, suggesting a role in β cell function (Smith et al., 2010; Soyer et al., 2010). Because of the postnatal lethality of *Rfx6*^−/−^ and Rfx6^ΔEndo^ mutants, we generated *Rfx6*^fl/fl^; Ins1-CreER^T2^ mice (called hereafter Rfx6^Δbeta^) to determine the role of Rfx6 in adult β cells. All subsequent experiments, to address the role of Rfx6 in adult β cells, were performed with 8- to 10-week-old mice (age of tamoxifen treatment). Ins1-CreER^T2^ mice specifically and efficiently delete floxed alleles in β cells upon tamoxifen treatment (Figure S2). Similarly, in Rfx6^Δbeta^ mice, *Rfx6* is efficiently deleted (95%) as early as 5 days after the first tamoxifen injection as shown by quantitative RT-PCR (qRT-PCR) on whole islets (Figures 2G and S2) and specifically in insulin-positive β cells (Figures 2A, 2B, and S2). Although *Ins1* transcripts were reduced by ∼54%–65% (Table S1; Figure 2H), but not *Ins2* (Table S1), insulin peptide, as well as c-peptide 1 and 2, are still detected in β cells lacking Rfx6 as late as 3 weeks after tamoxifen injections (Figures 2A–2F). In agreement with these observations, Rfx6^ΔBeta^ mice do not develop overt diabetes. Detection of insulin hormone in Rfx6^ΔBeta^ islets was surprising, because a previous study suggested that Rfx6 was necessary for insulin production in the embryonic pancreas (Smith et al., 2010). Using quantitative chromatin immunoprecipitation (ChIP) in Min6B1 cells (Lilla et al., 2003), we could not reveal any binding of Rfx6 to any of the eight predicted X-boxes in a 10 kb region upstream of *Ins1* gene, while in a control experiment, MafA mapped to the *Ins1* promoter region (not shown). Thus, induced inactivation of *Rfx6* in a mature β cell does not preclude the production of insulin hormone.

### Impaired Insulin Secretion and Glucose Intolerance in Rfx6^ΔBeta^ Mice

**Figure 3:**
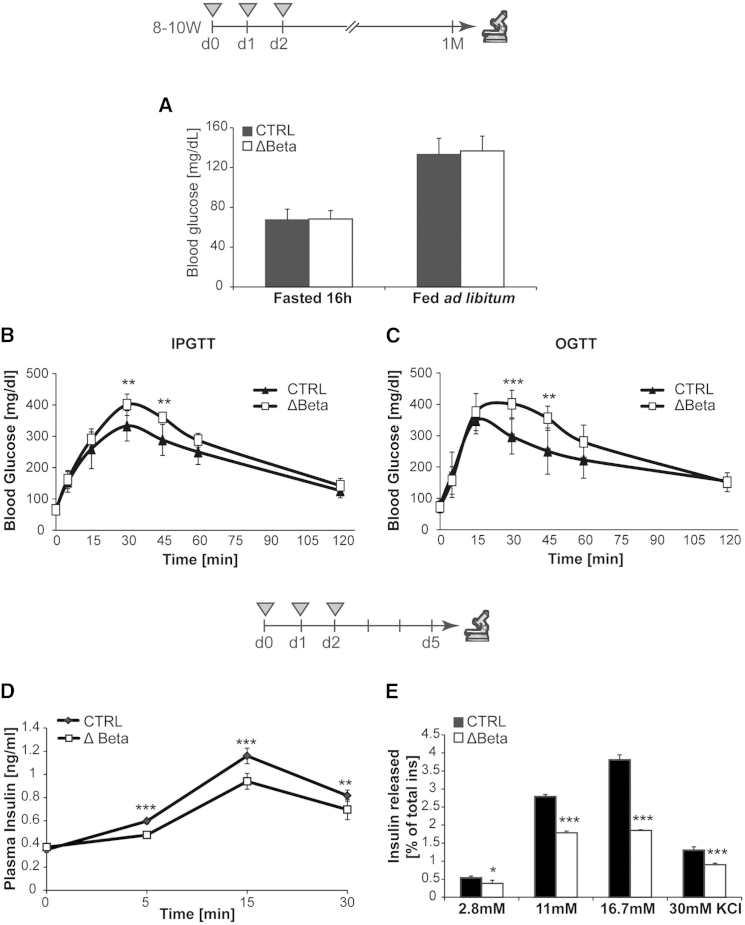
The Deletion of *Rfx6* in β Cells Causes Glucose Intolerance due to Defective Insulin Secretion (A–C) Exploration of glucose metabolism in adult (12- to 14-week-old) controls (n = 8) and Rfx6^ΔBeta^ (n = 7) males under normal diet 4 weeks after tamoxifen injections. Blood glucose levels are measured in overnight (16 hr)-fasted and ad libitum-fed animals 1 month after tamoxifen injections (A). Intraperitoneal glucose tolerance test (IPGTT) after 16 hr fasting in male mice 1 month after tamoxifen injections (B). Oral glucose tolerance test (OGTT) after 16 hr fasting in male mice 1 month after tamoxifen injections (C). (D) Histogram representing the plasma insulin levels of control and Rfx6^ΔBeta^ mice (9–11 weeks old) during an in vivo glucose-stimulated insulin secretion test (n = 6) performed 5 days after tamoxifen injections. (E) Histogram representing the insulin released during an ex vivo glucose- and KCl-stimulated insulin secretion tests on islets purified from controls and Rfx6^ΔBeta^ (n = 4) mice (9–11 weeks old), 5 days after tamoxifen injections. Data are presented as mean ± SD; ^∗^p < 0.05, ^∗∗^p < 0.01, ^∗∗∗^p < 0.001.

To determine whether glucose homeostasis was perturbed in Rfx6^ΔBeta^ mice, we measured the blood glucose concentration in fasted and ad libitum-fed mice and conducted intraperitoneal (i.p.) and oral (O) glucose tolerance tests (GTT) in 3-month-old males, 1 month after tamoxifen treatment. Basal blood glucose concentration was similar in Rfx6^ΔBeta^ and control mice when either fasted or fed (Figure 3A). After 16 hr fasting, glucose was administrated by either i.p. injection or intragastric gavage (2 g/kg body weight). In both conditions, mutant mice displayed significant glucose intolerance (Figures 3B and 3C). Impaired glucose homeostasis in Rfx6^ΔBeta^ mice appears not to be due to defects in β cell mass, α cell mass, or β cell proliferation under the same conditions (Figure S3). To determine whether the reduced glucose clearance resulted from altered insulin levels, we measured plasma insulin in Rfx6^ΔBeta^ and control mice 5 days after tamoxifen treatment (Figure 3D). In fasted animals, the plasma insulin level in Rfx6^ΔBeta^ and control mice was similar (mean controls = 0.35 ng/ml; mean mutants = 0.37 ng/ml; n = 7, p > 0.05). However, significant differences were observed following i.p. glucose injection (3 g/kg body weight), as glucose-induced insulin secretion was much less efficient in Rfx6^ΔBeta^ mice (Figure 3D). Both the first- and second-phase responses were reduced in mutant mice. To determine whether the defect was islet autonomous, we purified islets from Rfx6^ΔBeta^ and control adult mice under the same conditions and performed ex vivo glucose-stimulated insulin secretion (GSIS) and KCl-stimulated insulin secretion (KCl-SIS) studies (Figure 3E). The amount of secreted insulin was normalized to the total insulin content, which was identical in controls and mutants (not shown), confirming that Rfx6 is not key for insulin production in adult β cells. Clearly, the extent of insulin secretion in response to a glucose or KCl stimulus was reduced in mutant islets compared with controls. Notably, the altered KCl-induced response suggests that the defective insulin secretion might result from glucose-independent depolarization defects. These results suggest that impaired glucose clearance results from insulin secretory failure that we detect as early as 1 week after *Rfx6* deletion both in vivo and ex vivo (in islets).

### Rfx6 Binds to X-Boxes in the *Gck* and *Abcc8* Genes, and Their Expression Is Strongly Decreased in Rfx6^ΔBeta^ Islets

**Figure 4:**
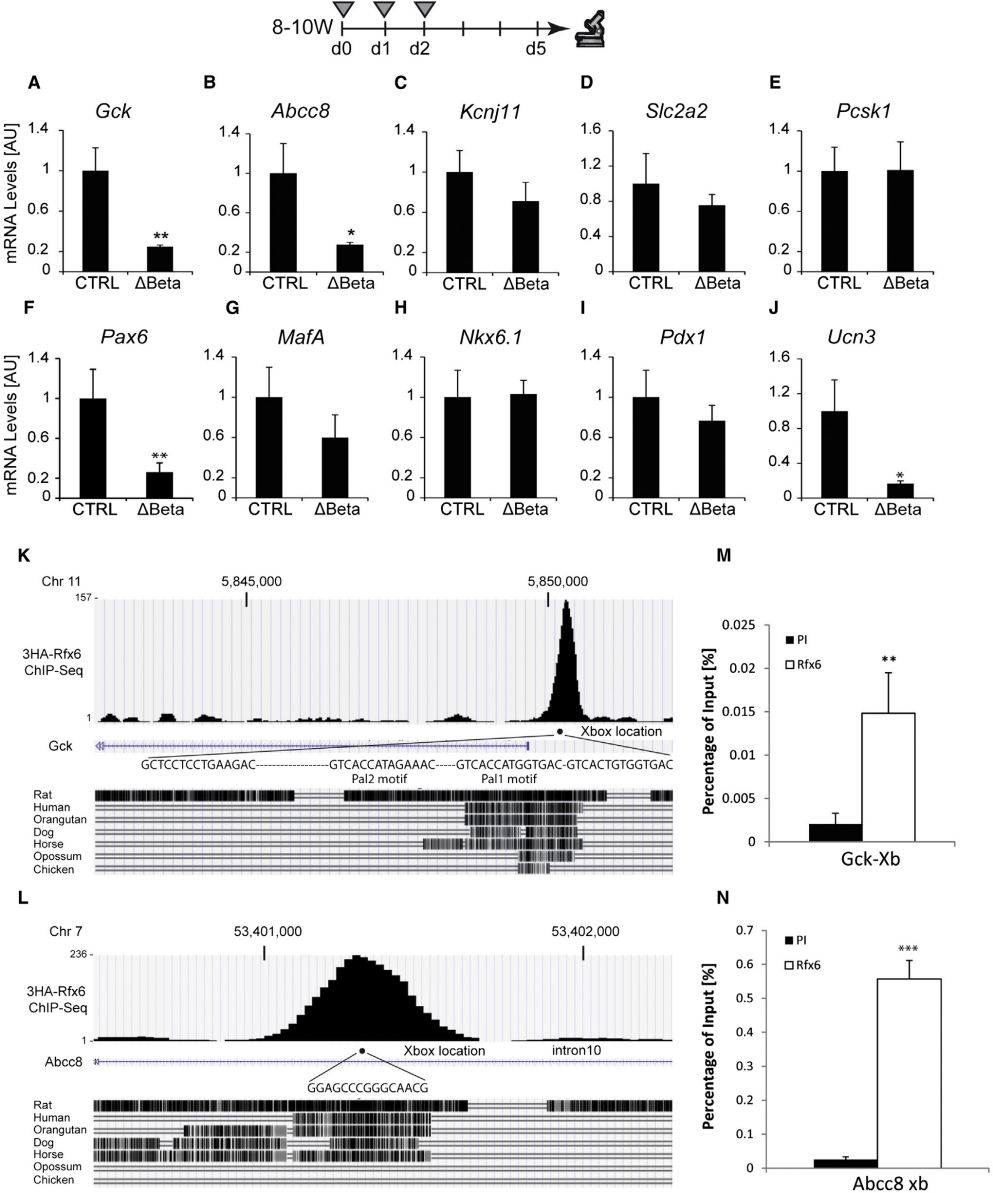
Rfx6 Regulates a Subset of Genes Controlling β Cell Function and Directly Targets *Gck* and *Abcc8* (A–J) qRT-PCR experiments for *Gck*, *Kcnj11*, *Abcc8*, *Slc2a2*, *Pcsk1*, *Pax6*, *MafA*, *Nkx6.1*, *Pdx1*, and *Ucn3* on islets purified from controls and Rfx6^ΔBeta^ adult mice (9–11 weeks old), 5 days after tamoxifen injections. Grey triangles indicate the days of tamoxifen injections. (K and L) ChIP-seq (anti-HA) data showing Rfx6 binding peaks in *Abcc8* and *Gck* genes in 3HA-Rfx6 transfected Min6B1 cells. (M and N) Quantitative ChIP (anti-Rfx6 antibody) in Min6B1 cells illustrating the binding of Rfx6 to X-boxes indicated in (K) and (L). PI and Rfx6 stand for preimmune and anti-Rfx6 serum. Data are presented as mean ± SD on n = 4–5 samples; ^∗∗^p < 0.01, ^∗^p < 0.05.

Next, our goal was to decipher the molecular basis underlying defective insulin secretion and glucose intolerance in Rfx6^ΔBeta^ mice. Therefore, we performed a series of qRT-PCR experiments to measure the expression of key regulators of glucose simulated insulin secretion in islets isolated from mutant or control mice, 5 days after tamoxifen treatment. Importantly, we found that transcripts encoding the key genes *Gck* and *Abcc8* were dramatically reduced (Figures 4A and 4B; Table S1). *Gck* encodes glucokinase, an enzyme that controls the first step of glycolysis and is considered the rate-limiting step in glucose metabolism (Matschinsky, 2005). *Abcc8/SUR1* encodes the regulatory sulphonylurea-binding subunits of the ATP-sensitive K^+^ channel (K_ATP_ channels) linking glucose metabolism to the electrical activity of β cells (McTaggart et al., 2010). Furthermore, *Ucn3* mRNA, a marker of β cell maturation and also reported to have a positive effect on GSIS (Li et al., 2007), is strongly decreased (Figure 4J). In contrast, the expression of the glucose-facilitated transporter *Slc2a*2/Glut2, the proconvertase *Pcsk1*, and the pore-forming unit of K_ATP_ channels (*Kcnj11*/Kir6.2) was not altered (Figures 4C–4E). Among the transcription factors important for β cell function and insulin transcription tested (*MafA*, *Pdx1*, *Nkx6.1*, *Pax6*, and *NeuroD1*), only *NeuroD1* and *Pax6* transcripts were downregulated (Figures 4F–4I; Table S1).

To determine whether Rfx6-regulated genes might be direct targets of Rfx6, we analyzed chromatin immunoprecipitation sequencing (ChIP-seq) experiments performed in the mouse β cell line Min6B1 (Lilla et al., 2003) transfected with hemagglutinin (HA)-tagged Rfx6. One peak was detected in the β cell-specific *Gck* promoter region (Figure 4K) overlapping with Pal1 and Pal2 regions previously demonstrated to contain X-boxes bound by Rfx3 (Ait-Lounis et al., 2010). Similarly, a peak was observed in a conserved region of intron 10 of the *Abcc8* gene (Figure 4L). Quantitative ChIP experiments in nontransfected Min6B1 cells, using an Rfx6 antibody, confirmed that endogenous Rfx6 binds to the *Gck* and *Abcc8* genes, although less strongly to *Gck* (Figures 4M and 4N). Taken together, our results suggest that Rfx6 is a direct positive transcriptional regulator of *Gck* and *Abcc8* and that the downregulation of these genes could impact glucose-stimulated insulin secretion.

### Glucose Fails to Induce Normal Increases in ATP/ADP Ratio and Intracellular Free Ca^2+^ in Rfx6^ΔBeta^ Mice

**Figure 5:**
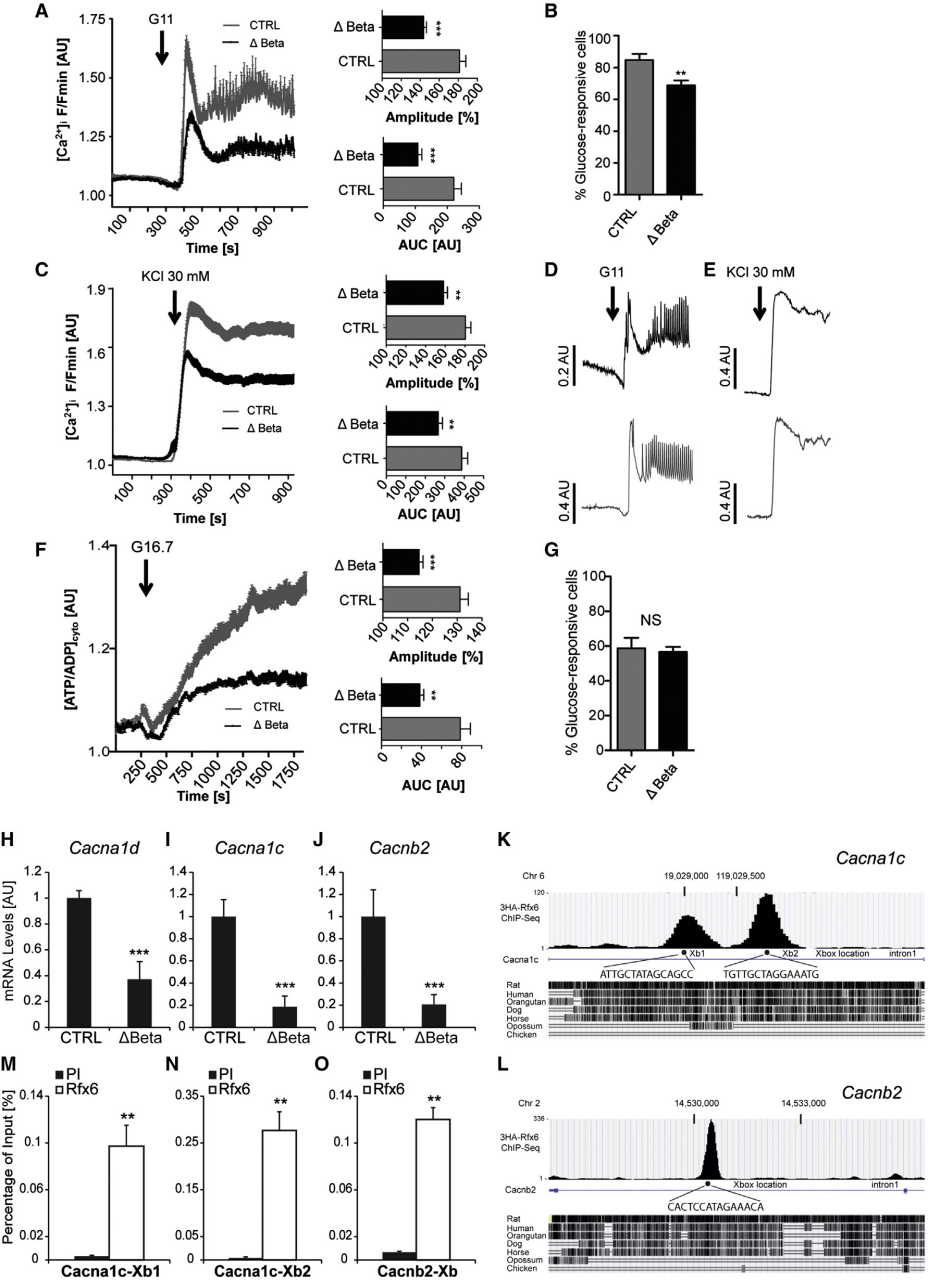
Impaired ATP/ADP Ratio and Calcium Trafficking in Glucose Stimulated β Cells in *Rfx6*^*ΔBeta*^ Islets (A) Mean (±SEM) Ca^2+^ traces following elevation of glucose from 3 mM to 11 mM (n = 16 islets from five mutants and six controls). Insets are the amplitude and area under the curves. (B) Proportion of fluo2-loaded cells that respond to 11 mM glucose. (C) Mean (±SEM) Ca^2+^ traces following application of the depolarizing stimulus 30 mM KCl (n = 18 islets from same animals as A). (D) Representative Ca^2+^ responses to 11mM glucose in a single islet (average of about 50 responsive cells per islet). (E) Representative Ca^2+^ responses to 30 mM KCl in a single islet. (F) Mean (±SEM) Perceval traces recording ATP dynamics following elevation of glucose from 3 mM to 16.7 mM (∼250 cells from n = 12 islets from four mutants and four controls). Insets are the amplitude and area under the curves of cytosolic ATP/ADP rises ([ATP/ADP]_cyto_). (G) Proportion of Perceval-expressing cells, shown to represent beta cells (Hodson et al., 2014a) that respond to 16.7 mM glucose. (H–J) qRT-PCR revealing the decrease in the transcription of *VDCC*s in Rfx6^ΔBeta^ islets (n = 4). (K–O) ChIP-seq (K and L) and quantitative ChIP (M–O) showing the binding of Rfx6 on X-boxes of *Cacna1c* and *Cacnb2* genes in Min6B1 cells (n = 3). PI and Rfx6 in (M) and (N) stand for preimmune and anti-Rfx6 serum. Data are presented as mean ± SD; ^∗∗∗^p < 0.001, ^∗∗^p < 0.01.

Given the abnormalities in glucose-stimulated insulin secretion and gene expression described above, we next explored the possibility that signal generation by the sugar may be impaired in Rfx6 null β cells. Examined using functional multicellular imaging within intact islets (Hodson et al., 2013, 2014a), both phases of the intracellular calcium ([Ca^2+^]i) response to a step increase in glucose concentration were sharply (50%–60%) reduced by *Rfx6* deletion (Figures 5A and 5D). A smaller (∼20%) reduction was also apparent in the overall number of responsive cells (Figure 5B). These changes were accompanied by more modest, but significant, defects in the response to depolarization with KCl (Figures 5C and 5E), suggesting a defect downstream of metabolic signal generation by glucose. Defects in the latter process were, nonetheless, highlighted by a substantial (∼60%) impairment in glucose-induced cytosolic ATP/ADP increases (Hodson et al., 2014b; Tarasov et al., 2012) (Figures 5F and 5G), consistent with lowered *Gck* expression.

**Figure 6:**
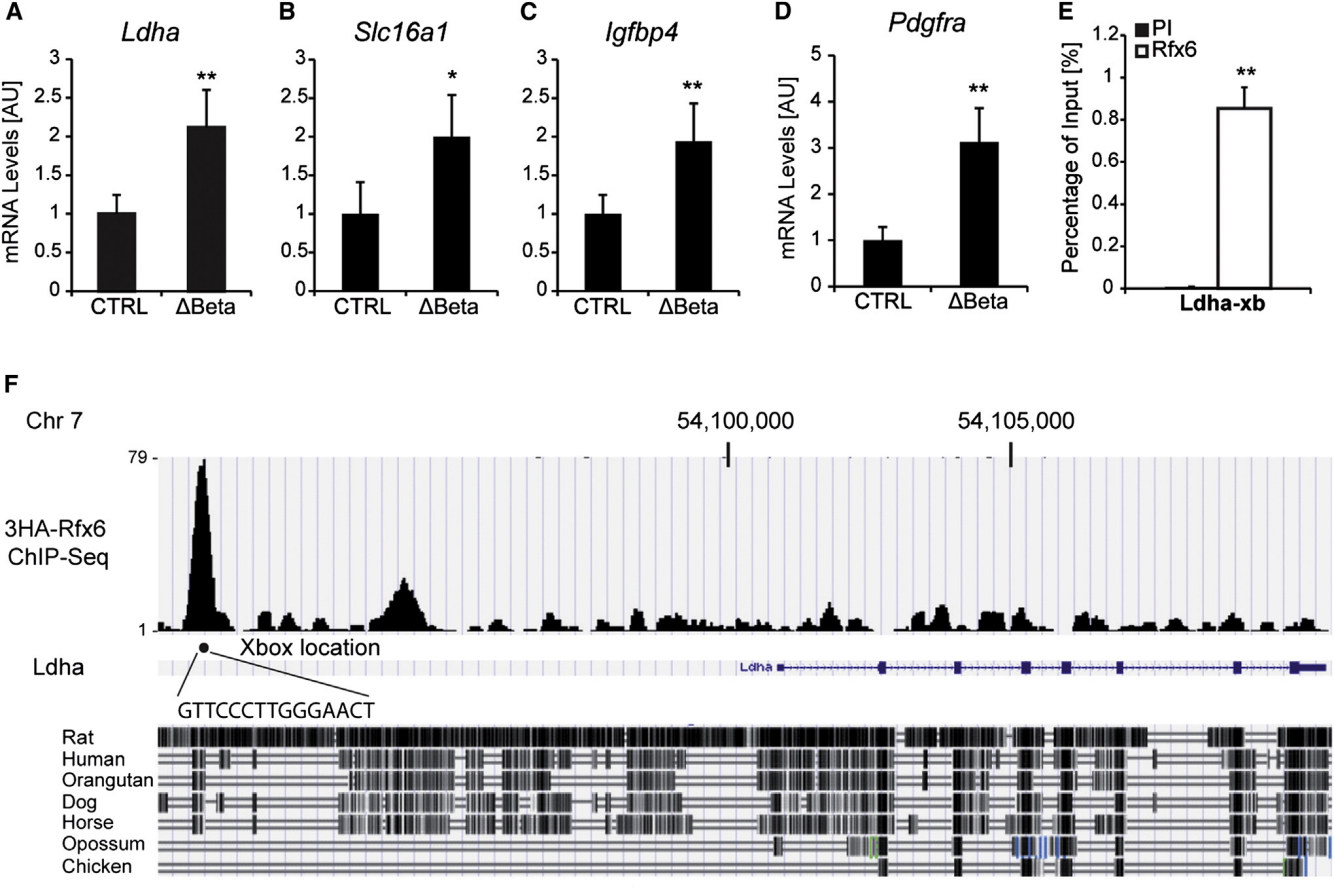
Rfx6 Targets and Represses Disallowed Genes in β Cells (A–D) qRT-PCR showing the downregulation of *Ldha*, *Slc16a1*, *Igfbp4*, and *Pdgfra* in islet cells from Rfx6^ΔBeta^ mice (9–11 weeks old), 5 days after tamoxifen injections compared to controls (n = 4). (E and F) ChIP-PCR and ChIP-seq revealing the binding of Rfx6 on one X-box in *Ldha gene* in Min6B1 cells (n = 3). Data are presented as mean ± SD; ^∗∗^p < 0.01, ^∗^p < 0.05.

In line with the above defects in calcium influx, RNA sequencing (RNA-seq) performed on Rfx6^ΔBeta^ and control whole islets, 5 days after tamoxifen treatment, revealed that the expression of eight voltage-dependent calcium channels (VDCCs) transcripts was downregulated by the deletion of *Rfx6* in adult β cells (Table S2). These genes included *Cacna1a*, *Cacnb2*, *Cacna1d*, and *Cacna1c*, which are among the most abundantly expressed VDCCs in rodent islets (Table S2). Downregulation of *Cacna1d*, *Cacna1c*, and *Cacnb2* was confirmed by RT-PCR (Figures 5H–5J). ChIP sequencing (ChIP-seq) in Min6B1 cells revealed that Rfx6 binds to two of the Rfx6-dependent VDCC genes, at X-boxes in intron 1 of *Cacna1c* and *Cacnb2* genes, respectively (Figures 5K and 5L), suggesting that Rfx6 can directly regulate these genes, a result confirmed by quantitative ChIP (Figures 6M–6O). Collectively, these data suggest that compromised insulin secretion in *Rfx6*-deficient β cells is the consequence, at least in large part, of the combined alteration of key steps in glucose signaling due to a failure of Rfx6 to directly promote the expression of key genes. These include both glycolysis, impaired by downregulation of *Gck*, and Ca^2+^ influx, affected by the repression of voltage-gated calcium channels.

### Derepression of the Disallowed Genes in β Cells Lacking Rfx6

We next sought to determine whether additional mechanisms may also play a role in defective glucose-induced insulin secretion in *Rfx6* null β cells. Rfx transcription factors have been reported to be either activators or repressors of transcription (Aftab et al., 2008). Accordingly, RNA-seq revealed a series of genes with a higher expression in β cells after *Rfx6* deletion. Surprisingly, we found a specific upregulation of the so-called disallowed or forbidden genes. Disallowed genes are genes that are abundantly expressed in most tissues but selectively repressed in adult mouse β cells. Two different studies identified a total of 68 of these genes by microarray analysis (Pullen et al., 2010; Thorrez et al., 2011). We found that 54 out of the 68 disallowed genes reported were upregulated in Rfx6^ΔBeta^ islets, including the 11 common genes identified in both studies (Pullen and Rutter, 2013), namely *Slc16a1*, *Ldha*, *Pdgfra*, *Igfbp4*, *Cxcl12*, *Oat*, *Smad3*, *Lmo4*, *C1qbp*, *Maf*, and *Cd302* (Table S3). We confirmed the upregulation of some of the selected genes by qRT-PCR: *Ldha*, *Slc16a1*, *Pdgfra*, and *Igfbp4* (Figures 6A–6D). ChIP-seq and quantitative ChIP in Min6B1 cells revealed that Rfx6 binds to a conserved region approximately 10 kb upstream of *Ldha* gene (Figures 6E and 6F), suggesting that Rfx6 could directly regulate the repression of this gene in β cells. Some of the disallowed genes have been reported to modulate insulin secretion (see Discussion and Pullen and Rutter, 2013). Thus, by repressing the disallowed genes, Rfx6 contributes to the maintenance of β cell identity and function.

## Discussion

Previous studies have revealed that Rfx6 is required during development in rodents and in humans for the generation of β cells (Smith et al., 2010). The present work now demonstrates that this factor is also essential for the maintenance of the functional identity of the adult β cell.

### Rfx6 and Insulin Production

To determine the role of Rfx6 in β cell function, we conditionally and specifically inactivated this factor in mature adult β cells. The resulting Rfx6^ΔBeta^ mice are glucose intolerant in contrast to the phenotype of the *Rfx6* null mice, which die shortly after birth with severe and sustained hyperglycemia (>600 mg/dl). This difference indicates that Rfx6 has a distinct function in adult β cells that is different from its earlier function in the embryonic pancreas. In the embryo, our data in Rfx6^ΔEndo^ mouse pancreas clearly show that Rfx6 is required downstream of Ngn3 for proper β cell development, when cells are already committed to an islet fate. It is unclear whether the strongly impaired expression of insulin transcripts and peptide in the Rfx6^ΔEndo^ embryonic pancreas reflects a blockade in the β cell differentiation program or a direct regulation of the transcription of *Insulin* genes by Rfx6. However, it is likely that Rfx6 has a very early function downstream of Ngn3 and implements genetic programs essential for islet subtype specification to proceed. This hypothesis is supported by the up- and downregulation of *Pax4* and *Arx*, respectively (also observed in the constitutive Rfx6 knockout; our own data and Smith et al., 2010), known to control β-δ versus α destiny (Collombat et al., 2003). One possibility would be that in a wild-type situation, Rfx6 would favor the α destiny by promoting *Arx* expression and simultaneously blocking the β fate by repressing *Pax4*. However, Rfx6 must have an additional role; otherwise, we would have observed an increased number of β cells in Rfx6^ΔEndo^ embryos, which is not the case. The maintenance of generic markers of endocrine differentiation such as chromogranin A testifies that endocrine differentiation proceeds in the absence of Rfx6. On the other hand, the strong downregulation of *Ins1*, *Ins2*, *Gcg*, *Ghr*, and *Sst* mRNAs suggests that Rfx6 implements a genetic program required for hormone production in embryonic islet cells. In adult mice, the overall production of insulin is not altered in Rfx6^ΔBeta^ islets and thus Rfx6 is not indispensible for insulin production in β cells. However, *Ins1* transcripts are decreased when *Rfx6* is deleted in β cells, while *Ins2* transcripts do not vary. A similar result has been reported in mice lacking *NeuroD1* in β cells (Gu et al., 2010), although *Ins1* was reduced by 95% in this model, in contrast to 65% in Rfx6^ΔBeta^ islets. ChIP-seq and quantitative ChIP in Min6B1 cells did not reveal any binding of Rfx6 to putative X-boxes, suggesting that Rfx6 does not directly regulate *Ins1* gene, although we cannot exclude that it is the case in bona fide β cells. Furthermore, like in mice lacking *NeuroD1* in β cells, the expression of other regulators of *Insulin* transcription such as *MafA*, *Nkx6.1*, and *Pdx1* is unchanged in Rfx6^ΔBeta^ islets, suggesting that the reduction in *Ins1* transcripts results from decreased levels of *NeuroD1*. Decreased *Ins1* transcript levels could also result from low *Pax6*, which is necessary for insulin synthesis in adult β cells (Hart et al., 2013). Importantly, we observed a downregulation, and not a complete loss, of *NeuroD1* and *Pax6* and suspect that levels of these transcription factors are sufficient to account for the insulin mRNA levels measured in Rfx6^ΔBeta^ islets. However, we did not observe any signal reduction in anti-insulin immunofluorescence experiments, and total insulin content was not affected either, probably because the reduction of *Ins1* mRNA was less severe compared to mice lacking *NeuroD1* in β cells. Such a decrease in *Ins1* transcripts without any significant effect on insulin content has been described previously, such as in *MafA*-deficient mice (Zhang et al., 2005), and may reflect alterations in mRNA translation.

### Rfx6 and Insulin Secretion

We found that without the transcription factor Rfx6, insulin secretion is impaired in β cells and mice become glucose intolerant. Defective insulin secretion resulted from a combined effect to reduce expression of three core components of the glucose-stimulated signaling pathway in β cells, which we show to be direct targets of Rfx6. First, mRNAs encoding the glucose sensor Glucokinase were decreased (∼75%) in Rfx6^ΔBeta^ islets. Glucokinase is the flux-generating enzyme of oxidative glycolysis, coupling blood glucose concentration to metabolic signals in β cells, ultimately leading to rises in cytosolic ATP/ADP ratios triggering the closure of K_ATP_ channels and subsequent membrane depolarization. Gene-deletion experiments in mice have demonstrated that Gck is essential for insulin secretion and maintenance of glucose homeostasis and that reduction in *Gck* gene dosage (Gck^+/−^ mice) is sufficient to induce mild hyperglycemia (Terauchi et al., 1995). We thus believe that decreased *Gck* levels are the principal cause of the failure of the cytosolic ATP/ADP ratio to increase appropriately upon glucose stimuli in Rfx6^ΔBeta^ islets. Interestingly, insulin secretion reaches a plateau at 11 mM glucose in Rfx6^ΔBeta^ islets, and further increases in glucose concentrations have no effect on insulin secretion, demonstrating that the response to glucose is saturated. Though other steps in the pathway may also be affected, this change in glucose dose response is consistent with a shift in the control of glycolytic flux toward other hexokinase family members (HKI-III) with lower Michaelis constants for the sugar. Second, we found that *Abcc8/SUR1* transcripts encoding the regulatory subunit of the ATP-sensitive K^+^ channel (K_ATP_ channels) are downregulated as well in Rfx6^ΔBeta^ islets. Thus, we propose that lowered *Abcc8*, which would tend to raise the resting plasma membrane potential at low glucose while impairing stimulation by the sugar (Nenquin et al., 2004), combined with suboptimal increases in ATP/ADP ratios, alters β cell membrane excitability. This, in turn, will affect the opening of voltage-dependent Ca^2+^ channels, at least partially explaining impaired glucose-induced Ca^2+^ influx. Third, we found that Rfx6 is also critical for the expression of several voltage-dependent calcium channels mRNAs of the L, R, and P/Q type, which are downregulated in Rfx6^ΔBeta^ islets. These include central players such as the L-type Ca^2+^ channel subunit alpha 1C (*Cacna1c*, also named as *Ca*_*v*_*1.2*) and the R-type Ca^2+^ channel alpha 1E (Cacna1e, also named Cav2.3) subunits, which have been shown to be essential for insulin secretion in mice by regulating first and second phases of secretion, respectively (Jing et al., 2005; Schulla et al., 2003). Thus, in addition to decreased glycolysis and reduced levels of K_ATP_ channel Abcc8/SUR1 subunits, lowered voltage-gated Ca^2+^ channels likely contributes to the impaired elevation of intracellular Ca^2+^ and accounts for defective KCl-stimulated insulin secretion. Finally, we noted that the zinc transporter *Slc30a8* (*ZnT8*), required for normal insulin crystallization and secretion (Rutter, 2010), was also repressed after *Rfx6* deletion (Table S1), suggesting that later events in the exocytotic process may also be affected. Nevertheless, and despite multiple deficiencies in several steps controlling glucose-induced secretion, Rfx6^ΔBeta^ are only mildly glucose intolerant. As we did not reveal any increase in β cell mass, we suggest that Rfx6^ΔBeta^ mice compensate their impaired insulin secretion by other mechanisms, which may include an increased insulin sensitivity or higher secretion of glucoincretins.

Importantly, ChIP experiments in the mouse β cell line Min6B1 suggest that Rfx6 binds to X-boxes in *Gck*, *Abcc8*, and the VDCC *Cacna1c* and *Cacnb2* genes. These results, together with the expression data of Rfx6^ΔBeta^ islets (RNA-seq), clearly suggest that Rfx6 controls β cell function in the adult by directly regulating the expression of key core components of the insulin-secretion pathway. Interestingly, *Gck* has been previously reported to be a direct target of Rfx3 (Ait-Lounis et al., 2010). The fact that we independently found, by an unbiased method (ChIP-seq), that Rfx6 binds to the very same region in the Gck promoter as Rfx3 strongly supports that Rfx transcription factors are important regulators of Gck expression. However, Rfx3 (*Rfx3* expression is unaffected in Rfx6^ΔBeta^ islets) was not sufficient to compensate for the absence of Rfx6, regarding *Gck* expression, suggesting that Rfx3-Rfx6 heterodimers bind to the *Gck* promoter for the optimal transcription of *Gck* gene. Binding of Rfx3 to *Abcc8* or voltage-dependent Ca^2+^ channel genes has not been reported, and although Rfx6 and Rfx3 might coregulate other targets genes, the extent of the functional redundancy of both transcription factors in β cell function and insulin secretion cannot be evaluated, as the phenotype of mice with a deletion of *Rfx3* in adult β cells has not been described. Likewise, whether a small but significant (∼30%) decrease in *Rfx5* expression (Table S1) observed here also contributes to the phenotype of *Rfx6* null adult mice remains a question for the future.

### Rfx6 Contributes to the Silencing of the Disallowed Genes and Maintenance of β Cell Maturity

A group of disallowed or “forbidden” housekeeping genes has recently been described to be selectively repressed in β cells. The potential role of their repression in β cell function and glucose homeostasis (discussed in detail in Pullen and Rutter, 2013) is still to be fully elucidated. However, it has been proposed for the two founder members, lactate dehydrogenase A (*Ldha*) and monocarboxylate transporter-1 (*MCT-1/Slc16a1*), that their downregulation in β cells prevents lactate and pyruvate, produced from muscle during exercise, from inappropriately stimulating insulin release (Thorrez et al., 2011). At present, it is unclear whether a general mechanism controls the silencing of all (or most) of the disallowed genes. However, epigenetic mechanisms such as the trimethylation of histone H3 on lysine 27 (H3K27me3) has been suggested to be the cause of disallowed gene repression, as H3K27me3 was found present on *Slc16a1* promoter (van Arensbergen et al., 2010) and a few other forbidden genes (*Cxcl12*, *Acot7*, *Nfib*, *Mgst1*, and *Maf*) (Pullen and Rutter, 2013). Likewise, DNA methylation is reported also to be involved in the repression of some (e.g., *Acot7*) (Dayeh et al., 2014), but not other (*Slc16a1*/*MCT1*) (Pullen et al., 2011), members of this family. On the other hand, microRNAs have also been reported to downregulate *Slc16a1* (Pullen et al., 2011). Our current study provides evidence of another mechanism whereby a transcription factor (Rfx6) may act as a master regulator, repressing disallowed genes and serving as a common mechanism for disallowed gene downregulation, as we found that ∼79% of these genes were significantly upregulated in Rfx6^ΔBeta^ islets. We found that Rfx6 binds to an X-box located ∼10 kb upstream of *Ldha* start site in Min6B1 cells, supporting direct repression. Along the same lines, and further supporting the notion that Rfx6 directly targets disallowed genes, we identified Rfx6 binding peaks in the vicinity of 32 out of 54 (59%) Rfx6-repressed disallowed genes (Table S3). Of note, increased expression of *Ldha* has also been described when *NeuroD1* is deleted in β cells (Gu et al., 2010). We thus cannot exclude that the repression of *NeuroD1*, observed in Rfx6^ΔBeta^ islets, also contributes to elevated *Ldha* transcript levels, a feature of neonatal β cells, in contrast to mature β cells, which have low amounts of *Ldha* (Gu et al., 2010; Sekine et al., 1994). Importantly, other members of the disallowed family have not been reported to be dysregulated in *NeuroD1*-deficient β cells. Taken together, our own and others’ data suggest that repression of disallowed genes is achieved by multiple mechanisms including transcription factor-mediated repression of gene expression. Tissue-specific gene repression is thought to proceed during the maturation of islet cells during postnatal stages (Thorrez et al., 2011), a period when β cells acquire a mature GSIS (Blum et al., 2012). Interestingly, we did not observe any change in the expression of disallowed genes in the embryonic pancreas of *Rfx6*-deficient mice (data not shown). Thus, we propose that Rfx6 is essential to establish and maintain the repression of the disallowed genes and thereby the maturity of β cells. The extent to which perturbation of disallowed gene expression contributes to the phenotype of Rfx6^ΔBeta^ mice remains to be studied. However, it was shown previously that overexpression of *Ldha* diminishes glucose-stimulated insulin secretion in islets (Ainscow et al., 2000; Ishihara et al., 1999). Moreover, forced overexpression of *Slc16a1* in β cells leads to insulin secretion in response to muscle-derived pyruvate (Pullen et al., 2012) and is thought to be responsible for exercise-induced hyperinsulinism in humans (Otonkoski et al., 2007). In addition to *Ldha* and *Slc16a1*, several other disallowed genes potentially impact insulin secretion and glucose homeostasis, acting as second messengers controlling insulin secretion or regulating trafficking of insulin granules (see Pullen and Rutter, 2013 for a review). Thus, Rfx6 controls β cell maturity and glucose homeostasis by repression of the disallowed genes and activation of core component of the insulin secretion pathway, including glucokinase, the Abcc8/SUR1 subunit of K_ATP_ channels and voltage-gated Ca^2+^ channels.

We show here that Rfx6 is required for normal β cell identity, sustaining the expression of signature β cell genes (*Gck*, *Abcc8*) and repressing that of disallowed genes. These findings raise the possibility that changes in RFX6 expression may contribute to β cell failure in type 2 diabetes (T2D) in humans. To date, however, no such changes in *RFX6* mRNA have been reported in T2D versus healthy donor β cells (Marselli et al., 2010; Taneera et al., 2012), despite the increased expression of 15 disallowed genes in human T2D (Pullen and Rutter, 2013). However, we would point out that these studies involved a relatively small number of subjects, and therefore, we need to wait for the results of much larger ongoing trials to conclude whether subtle changes in *RFX6* levels are associated with T2D. Furthermore, changes in RFX6 activity resulting from altered subcellular localization or posttranslational modifications cannot be excluded in diseased islets. Interestingly, fasting hyperglycemia has been reported in a patient bearing a heterozygote mutation in the RFX6 gene and SNP susceptibility to T2D, supporting a role for RFX6 in β cell function in humans (Artuso et al., 2014).

Finally these results suggest that, in addition to having fewer β cells, sufferers of neonatal diabetes who carry *RFX6* mutations are also likely to have defects in those cells that remain (Mitchell et al., 2004). Whether these individuals may therefore be susceptible to pharmacological treatments, including sulphonylureas or GLP-1 analogs, which could prompt insulin release from the remaining β cells, may be worthy of investigation.

## Experimental Procedures

### Immunostaining and Morphometric Analysis of Islets

Pancreata were fixed with 4% paraformaldehyde, cryo- or paraffin embedded, and stained with the primary and secondary antibodies listed in Supplemental Experimental Procedures. Antigen retrieval was used prior to staining for Rfx6. For bromodeoxyuridine (BrdU) detection assays, BrdU (50 mg/kg body weight) was injected 24 hr before sacrifice to assess proliferation in adult β cells. For α and β cell mass, quantification was performed every 2 mm and calculated as described in Figure S3. Four animals of each genotype were analyzed. Animal experiments were supervised by G.G. (agreement N°C67-59 approved by the Direction des Services Vétérinaires, Strasbourg, France) in compliance with the European legislation on the care and use of laboratory animals.

### Insulin Secretion Assay

For each animal (n = 4 per genotype, 8–10 weeks old), quadruplicates of five starved islets were placed in Eppendorf tubes containing 2 ml of Kreb’s buffer containing 2.8 mM glucose, 11 mM glucose, 16.8 mM glucose, or 2.8 mM glucose + 30 mM KCl and incubated for 1 hr, and the supernatant was collected to measure insulin secretion. Quadruplicates of five unstimulated islets were sonicated and extracted by acid-ethanol. Insulin in supernatants and islet lysates was measured by ELISA (ultrasensitive insulin ELISA, ALPCO). Secreted insulin was then normalized with total lysate insulin content and expressed as a percentage of total insulin.

To measure insulin secretion in vivo, 9- to 11-week-old males were fasted for 16 hr, and we collected blood from animals (n = 6 per genotype) before glucose injection and 5, 15, and 30 min after glucose injection. D-glucose solution (15%) was injected intraperitoneally at 3 g/kg body weight. Plasma was separated from blood by centrifugation, and circulating insulin was measured by ELISA (ultrasensitive insulin ELISA, ALPCO).

### RNA Sequencing

Total RNA was extracted from adult islets from three controls and three mutants, and RNA integrity was assessed. After sequencing (Hiseq 2500, 50 base reads), reads were mapped onto the mm9 assembly of the mouse genome by using Tophat v1.4.1 (Trapnell et al., 2009) and the bowtie v0.12.7 aligner (Langmead et al., 2009). Only uniquely aligned reads have been retained for further analyses.

### Calcium and ATP Imaging

Functional multicellular Ca^2+^-and ATP/ADP-imaging were performed as previously described (Hodson et al., 2013; Hodson et al., 2014a, 2014b; Tarasov et al., 2012). Briefly, fluo2-loaded or Perceval-expressing islets were mounted in a custom-manufactured aluminum heated chamber and perifused with a HEPES-bicarbonate buffer (120 mM NaCl, 4.8 mM KCl, 24 mM NaHCO_3_, 0.5 mM Na_2_HPO_4_, 5 mM HEPES, 3 mM D-glucose, 2.5 mM CaCl_2_, and 1.2 mM MgCl_2_) saturated with 95% O_2_/5% CO_2_ and adjusted to pH 7.4. During recording, islets were maintained at 36°C and drugs/glucose delivered through the perfusion system at the indicated concentrations. Excitation was performed using a 491 nm solid-state laser (Cobalt) coupled to a Yokogawa CSU10 Nipkow spinning disk head, and emitted signals were captured at 525 ± 50 nm using a Hamamatsu 16-bit electron-multiplying charge-coupled device. An adenoviral vector was used to deliver cDNA encoding Perceval into the first few islet layers (multiplicity of infection, 10–100; 48 hr incubation).

### Statistics

Values are presented as mean ± SD, and p values were determined using the two-tailed Student’s t test with unequal variance. p < 0.05 was accepted as statistically significant.

## Author Contributions

J.P. designed the experiments, acquired and analyzed the data, and wrote the manuscript; P.S. performed the Rfx6 ChIP on Min6B1 cells; A.M. participated in the acquisition and analysis of the data; D.J.H. performed and analyzed the ex vivo calcium and ATP imaging experiments; T.Y. analyzed ChIP-seq data; C.K. analyzed RNA-seq data; M.C.B. generated and characterized Ins1-CreERT2 mice; and G.G. and G.A.R. designed the experiments, interpreted the data, and wrote the manuscript.

## Accession Numbers

RNA-seq and ChIP-seq data have been deposited to the Gene Expression Omnibus under the accession numbers GSE59622 and GSE62844.

## Supplemental Information

Document S1. Supplemental Experimental Procedures, Figures S1–S3, and Tables S1–S3

Document S2. Article plus Supplemental Information
