# Supplementary material for: Rfx6 Maintains the Functional Identity of Adult Pancreatic β Cells
Source: Cell Rep. 2014 Dec 11;9(6):2219–32. doi: 10.1016/j.celrep.2014.11.033 (PMC4542305; doi:10.1016/j.celrep.2014.11.033)
Supplement: Document S1. Supplemental Experimental Procedures, Figures S1–S3, and Tables S1–S3 [file mmc1.pdf]

Cell Reports, Volume *9*

Supplemental Information

Rfx6 Maintains the Functional Identity of Adult Pancreatic β Cells

Julie Piccand, Perrine Strasser, David J. Hodson, Aline Meunier, Tao Ye, Céline Keime, Marie-Christine Birling, Guy A. Rutter, and Gérard Gradwohl

Supplemental Experimental Procedures

Mouse strains, genotyping and manipulations

Animal experiments were supervised by G. Gradwohl (agreement N°C67-59 approved by the Direction des Services Vétérinaires, Strasbourg, France) in compliance with the European legislation on care and use of laboratory animals. Mice were maintained on C57/B6J genetic background on a 12/12 hour light/dark cycle with unlimited access to water and standard rodent chow diet. Ngn3-Cre mice are a gift from Dr. Shosei Yoshida (Yoshida et al., 2004), Ins1-CreER^{T2} mice were generated at the ICS (Illkirch, France) in the context of the CreER Zoo. Rfx6 Floxed mice were generated in collaboration with the ICS. Genomic tail DNA was analyzed by PCR using the primers described below. Rfx6$^{fl/fl}$; Ngn3-Cre (Rfx6$^{\Delta Endo}$) were used for analysis of the endocrine phenotype and both Rfx6$^{fl/fl}$ or Rfx6$^{fl/+}$ littermates were used as controls. Rfx6$^{fl/fl}$; Ins1-CreER^{T2} (Rfx6$^{\Delta Beta}$) and Rfx6$^{fl/fl}$ (control) 8-10 weeks old mice were both injected subcutaneously with 100µl (1mg) of tamoxifen (Sigma, dissolved in filtered corn oil at 10mg/ml) daily during 3 days. The mice were analysed 5 days, 3 weeks or 1 month after the first injection. Note that only males were used for metabolic experiments whereas a mixture of male and females was used for transcripts analysis.

Mouse Islet Purification

Mouse islet purification was performed as previously described (Piccand et al., 2014). Briefly, mice were euthanized and injected with Type V Collagenase (Sigma C9263) solution directly into the common bile duct to perfuse the pancreas. Pancreas was dissected out and digested and islets were handpicked after several purification steps and used for RNA isolation or kept in culture over night before insulin secretion tests.

Glucose tolerance tests

For Oral Glucose Tolerance Test (OGTT), 16-hours fasted 12 weeks-old males received glucose by intragastric gavage (2g/kg body weight of 15% D-glucose). For Intraperitoneal Glucose Tolerance Test (IPGTT), 16-hours fasted 13 weeks-old males received glucose by intraperitoneal injection (2g/kg body weight of 15% D-glucose). For both GTT, circulating blood glucose was measured in tail blood at 0, 5, 15, 30, 45, 60 and 120 minutes using Accu-Check Performa (Roche).

Real time PCR analysis

Total RNA from newborn pancreas or purified islets was extracted using TRI Reagent (Invitrogen). Eventual genomic DNA contamination was removed by DNase I digestion (Roche). cDNA was synthesized using random primers (Roche) and Transcriptor Reverse Transcriptase (Roche). Quantitative PCR was carried out (LightCycler 480, Roche) with either Taqman probes (Applied Biosystems) or UPL probes and primers (Roche) as listed below. Gene expression levels were normalised to Rplp0.

ChIPSeq and ChIP-qPCR in Min6B1 cells

Min6B1 cells were grown in DMEM with 25mM glucose supplemented with 15% foetal calf serum, penicillin/streptomycin and 71µM β-mercaptoethanol, in a 5% CO2 incubator at 37°C. Cells were transfected with pCMV-Tag2A-3HA-Rfx6 plasmid using Lipofectamine 2000 (Invitrogen) and harvested two days later. For ChIP, after removing the medium, cells (transfected and untransfected Min6B1) were cross-linked for 10min at room temperature with 1% formaldehyde. The reaction was quenched with 125mM glycine and cells were washed with PBS and harvested by scrapping and centrifugation. Cells were resuspended in buffer I (25mM Tris HCl pH8; 1.5mM MgCl2; 10mM KCl; 0.1% NP40 (Sigma-Aldrich); Protease inhibitor cocktail (Roche)) using a Dounce homogenizer. After centrifugation, the pellet was resuspended in buffer II (1%SDS ; 10mM EDTA ; 50mM Tris HCl pH8 Protease inhibitor cocktail (Roche)). Chromatin was then sonicated using a Covaris E210 (USA) to generate chromatin fragments of 500 bp. Cell debris were removed by centrifugation at 11000g for 10min. Each ChIP was performed with 50µg of chromatin (4.10^6 cells) and a 10% aliquot was removed for the input control. Samples were pre-cleared with Protein-G-sepharose beads and next anti-HA antibody (12CA5, IGBMC) was added and incubated overnight at 4°C in buffer III (16.7mM Tris HCl pH8 ; 0.01% SDS ; 1.1% Triton ; 1.2mM EDTA ; 167mM NaCl ; Protease inhibitor cocktail (Roche)). The following day, Protein-G-sepharose beads were added during 4h. After washes in buffer IV and V (20mM Tris HCl pH8; 0.1% SDS; 1% Triton; 2mM EDTA; 150mM (IV) or 500mM (V) NaCl; Protease inhibitor cocktail (Roche)) and finally in Tris-EDTA. Bound DNA was eluted in elution buffer (1%SDS, 100mM NaHCO3) and reverse-crosslinked by proteinase K treatment overnight at 65°C. DNA was purified the following day by standard phenol-chloroform extraction. Briefly ChiPSeq was performed using an HiSeq 2500 (Illumina) sequencer and peak detection was performed using the MACS

software (Zhang et al. 2008). Peaks were then annotated using GPAT on the mouse genome (mm9). Ref Seq genes, genomic features and corresponding coordinates were downloaded from the USCC genome browser. Selected Rfx6 targets identified by ChIPSeq were confirmed by ChIP-qPCR (LightCycler 480, Roche) to determine the relative enrichment. The later experiments were performed on non-transfected Min6B1 cells using anti-Rfx6 (2767, IGBMC), and preimmune (2767, IGBMC) sera and appropriate UPL probes and primers (Roche) as listed below. ChIPSeq Data have been deposited at the Gene Expression Omnibus depository (GEO, Accession number GSE62844).

SUPPLEMENTAL REFERENCES

Piccand, J., Meunier, A., Merle, C., Jia, Z., Barnier, J.V., and Gradwohl, G. (2014). Pak3 promotes cell cycle exit and differentiation of beta-cells in the embryonic pancreas and is necessary to maintain glucose homeostasis in adult mice. Diabetes *63*, 203-215.

Yoshida, S., Takakura, A., Ohbo, K., Abe, K., Wakabayashi, J., Yamamoto, M., Suda, T., and Nabeshima, Y. (2004). Neurogenin3 delineates the earliest stages of spermatogenesis in the mouse testis. Developmental biology *269*, 447-458.

Zhang Y, Liu T, Meyer CA, Eeckhoute J, Johnson DS, Bernstein BE, Nusbaum C, Myers RM, Brown M, Li W, Liu XS. (2008). Model-based analysis of ChIP-Seq (MACS). Genome Biol.;9(9):R137.

Primary and Secondary Antibodies used in this study

Antibody Name	Vendor/Provider	Dilution
Guinea pig@Insulin	Linco (4011-01F)	1/1000
Mouse@Insulin	Sigma (I-2018)	1/1000
Guinea pig@Glucagon	Linco (4031-01F)	1/2000
Guinea pig@PP	Linco (4041-01F)	1/1000
Rat@Somatostatin	Chemicon (MAB354)	1/500
Goat@chromograninA	Santa Cruz (sc-1488)	1/500
Rabbit@Rfx6	IGBMC (2766)	1/500
Rabbit@MafA	Bethyl (A300-611A)	1/2000
Rabbit@Pdx1	Dr. Chris Wright (Vanderbilt University)	1/2000
Rabbit@Glut2	Chemicon (AB1342)	1/500
Rabbit@C-peptide 1	BCBC (AB1044)	1/2000
Rabbit@C-peptide 2	BCBC (AB1042)	1/3000
Rat@BrdU	AbD Serotec (OBT0030S)	1/10
Donkey@rabbitDyLight549	Jackson Immuno Research (711-505-152)	1/500
Donkey@gpDyLight488	Jackson Immuno Research (706-486-148)	1/500
Donkey@goatDyLight549	Jackson Immuno Research (705-505-147)	1/500
Donkey@ratDyLight488	Jackson Immuno Research (712-485-153)	1/500
Donkey@gpDyLight549	Jackson Immuno Research (706-505-148)	1/500
Donkey@mouseDyLight488	Jackson Immuno Research (715-485-150)	1/500
Donkey@rat biotinylated	Jackson Immuno Research (712-065-153)	1/500
Streptavidin-Alexa488	Molecular Probes (S-11223)	1/500

Primers or TaqMan and UPL probes used in this study

Gene	Forward primer or TaqMan ID	Reverse primer	UPL Probe	Application
Ngn3-Cre	ctgcagtttagcagaacttcagaggga	atcaacgttttgttttcgga	NA	Genotyping
ERT	gcattaccggtcgatgcaacgagtgatgag	aggatctctagccaggcaca	NA	Genotyping
Rfx6 flox	gaaggtgcacccataaaagc	tataagccacccagggtcag	NA	Genotyping
Cacnb2-xb	cctgtttgttttgctcctagc	gaaggcagctggggaacta	#33	ChIP-PCR
Cacna1c-xb1	gcttgctgtctcctgagtttc	cattactgcatttcctagcaacac	#67	ChIP-PCR
Cacna1c-xb2	gctcttggcttgctgctaac	tttgtggggaaagcagagat	#105	ChIP-PCR
Ldha-xb	tgtcctctgtcagccatcag	aagcagaaaaagcaacaacga	#41	ChIP-PCR
Abcc8-xb	tgaaagagccctggtgttttat	gtatgtatacaaccagcctggaaa	#42	ChIP-PCR
Gck-xb	ggtcaccatagaaaccacagg	caaccaggtgtggagtagatgtc		ChIP-PCR
Rfx6	tgcaggaaagagaaactggag	ggaaatttttggcgaattgtc	#83	RT-qPCR
MafA	ctccagagccaggtggag	gtacaggtcccgctccttg	#10	RT-qPCR
Ucn3	gacctgagcatttccactcc	cagaagtggcagcaggaagt	#105	RT-qPCR
Cacna1d	gaagctgcttgaccaagttgt	aacttccccacggttacctc	#9	RT-qPCR
Cacna1c	ccaacctcatcctcttcttca	acatagtctgcattgcctaggat	#71	RT-qPCR
Cacnb2	gcaggagagccagatgga	tcctggctccttttccatag	#67	RT-qPCR
Ldha	ggcactgacgcagacaag	tgatcacctcgtaggcactg	#12	RT-qPCR
ChgA	Mm00514341_m1		NA	RT-qPCR
Ins1	Mm01259683_g1		NA	RT-qPCR
Gck	Mm00439129_m1		NA	RT-qPCR
Abcc8	Mm00803450_m1		NA	RT-qPCR
Kcnj11	Mm00440050_s1		NA	RT-qPCR
Slc2a2	Mm00446228_m1		NA	RT-qPCR
Pcsk1	Mm00479023_m1		NA	RT-qPCR
Gcg	Mm00801712_m1		NA	RT-qPCR
Ppy	Mm00435889_m1		NA	RT-qPCR
Sst	Mm00436671_m1		NA	RT-qPCR
Arx	Mm00545903_m1		NA	RT-qPCR
Pax4	Mm01159036_m1		NA	RT-qPCR
Pdx1	Mm00435565_m1		NA	RT-qPCR
Nkx6.1	Mm00454962_m1		NA	RT-qPCR
Insm1	Mm02581025_s1		NA	RT-qPCR
MafB	Mm0062748_s1		NA	RT-qPCR
NeuroD1	Mm00520715_m1		NA	RT-qPCR
Pax6	Mm00443081_m1		NA	RT-qPCR
Ngn3	Mm00437606_s1		NA	RT-qPCR
Rplp0	Mm01974474_gH		NA	RT-qPCR

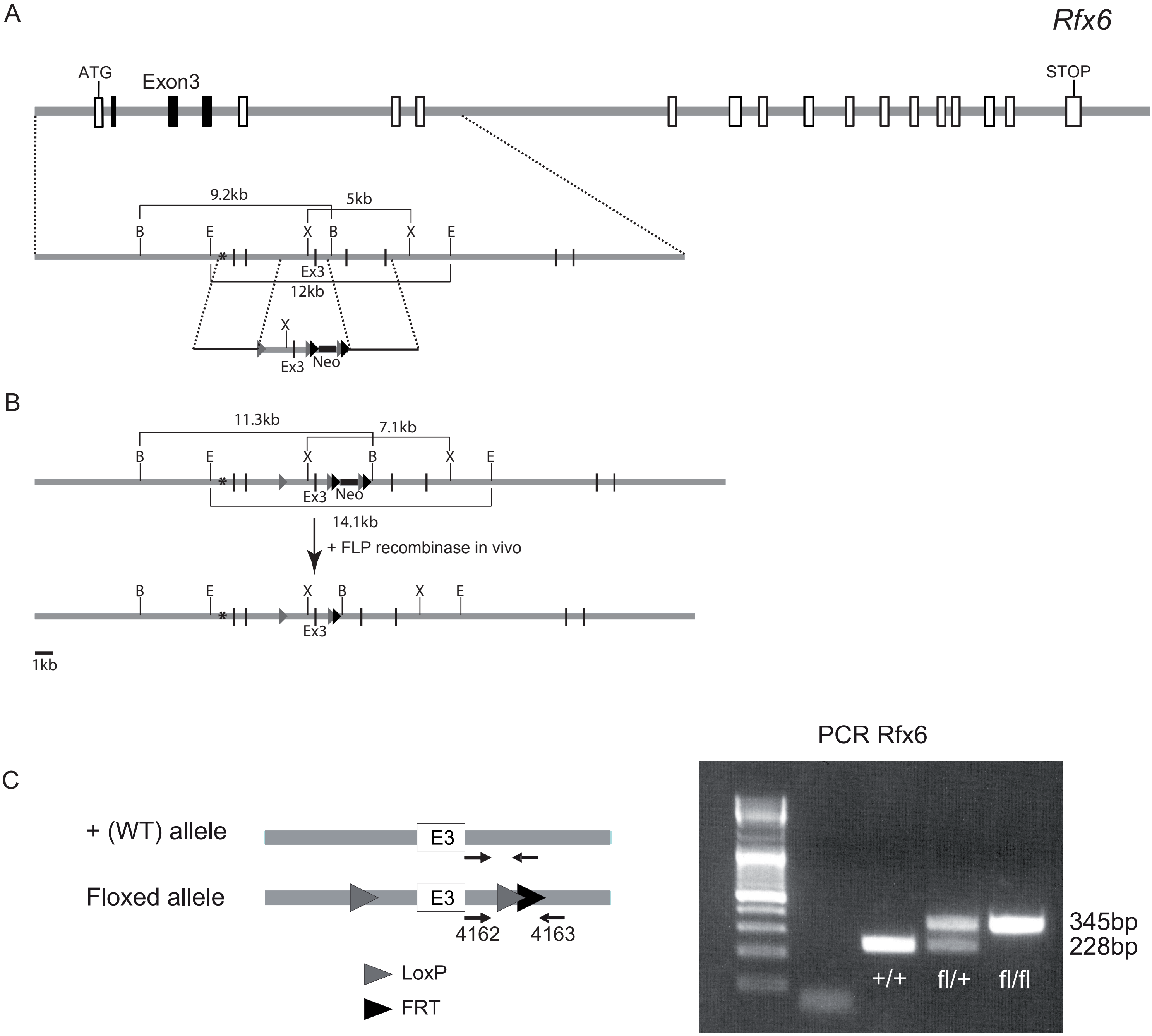

Figure S1: Generation of mice with a conditional Rfx6 allele used in this study related to Experimental Procedures.

(A) Schema depicting the mouse *Rfx6* locus and targeting construct. The DNA binding domain is composed of the exons represented by black boxes. (B) Targeted *Rfx6* allele before and after the excision of the FRT flanked “Neo” selection cassette by the FLP recombinase. (A-B) Star indicates the position of the 5’- external probe used for Southern blot analysis. In B, the exons are represented as thick black bars. The Neomycin selection cassette is the black box, loxP and FRT sites are the grey and black triangles respectively. B: BsaBI, E: EcoNI, X: XhoI. Scale is 1kb. (C) Genotyping strategy. Primers were designed to discriminate WT and floxed alleles (Supplemental Experimental Procedures). In the PCR gel on the right, the Rfx6 floxed allele generates a band at 345bp and the WT allele at 228bp. Grey triangles represent loxP sites and the black triangle represents the remaining FRT site.

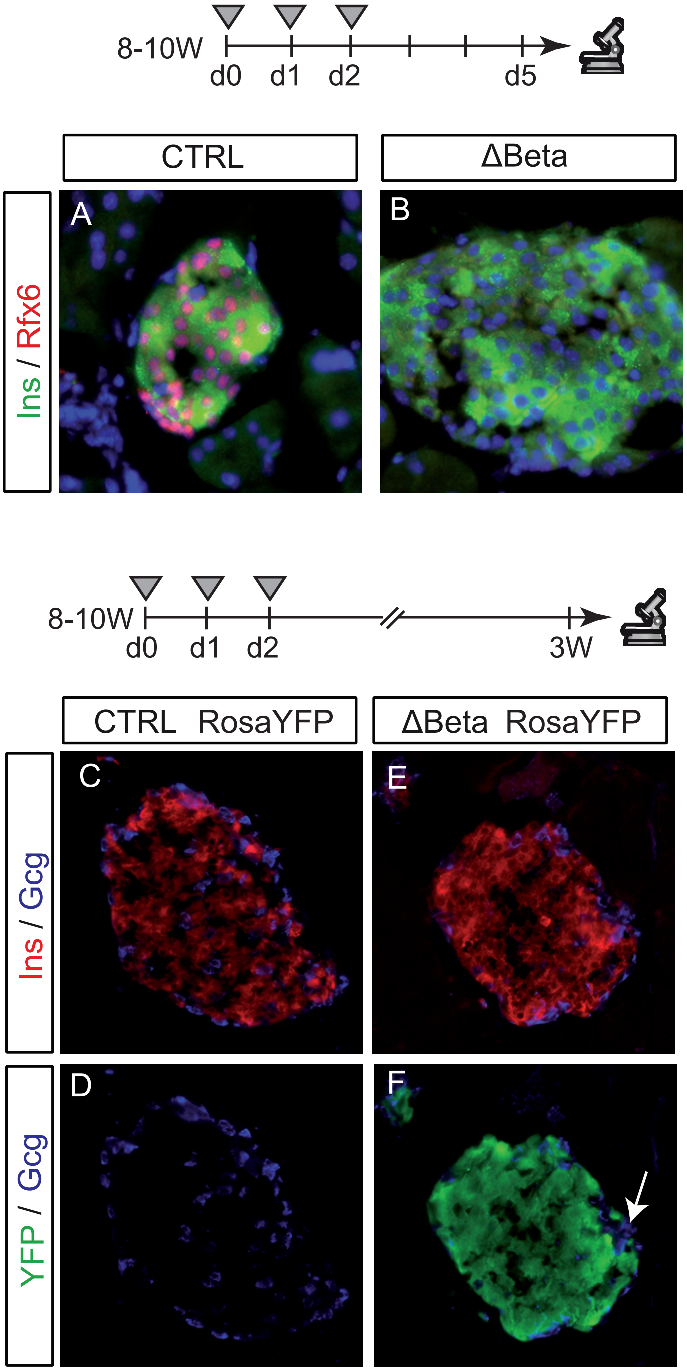

Figure S2: Data showing the efficient and specific deletion of Rfx6 in β-cells of ΔBeta mice upon tamoxifen injections related to Figure 2. (A-B) Rfx6 immunostaining is lost in insulin(Ins)-positive β-cells in ΔBeta mice (Rfx6fl/fl; Ins1-CreERT2) 5 days after tamoxifen treatment in contrast to control mice (Rfx6fl/fl). Grey triangles indicate the days of tamoxifen injections. (C-F) Tracing of the Cre activity of Ins1-CreERT2 mice in CTRL and ΔBeta mice crossed with the Cre reporter line RosaYFP. Strong YFP immunofluorescence in β-cells of ΔBeta; RosaYFP mice (F) after tamoxifen treatment in constrast to control mice (D). Note that as expected α-cells (labeled by Glucagon, Gcg) are note marked by YFP (arrow in F).

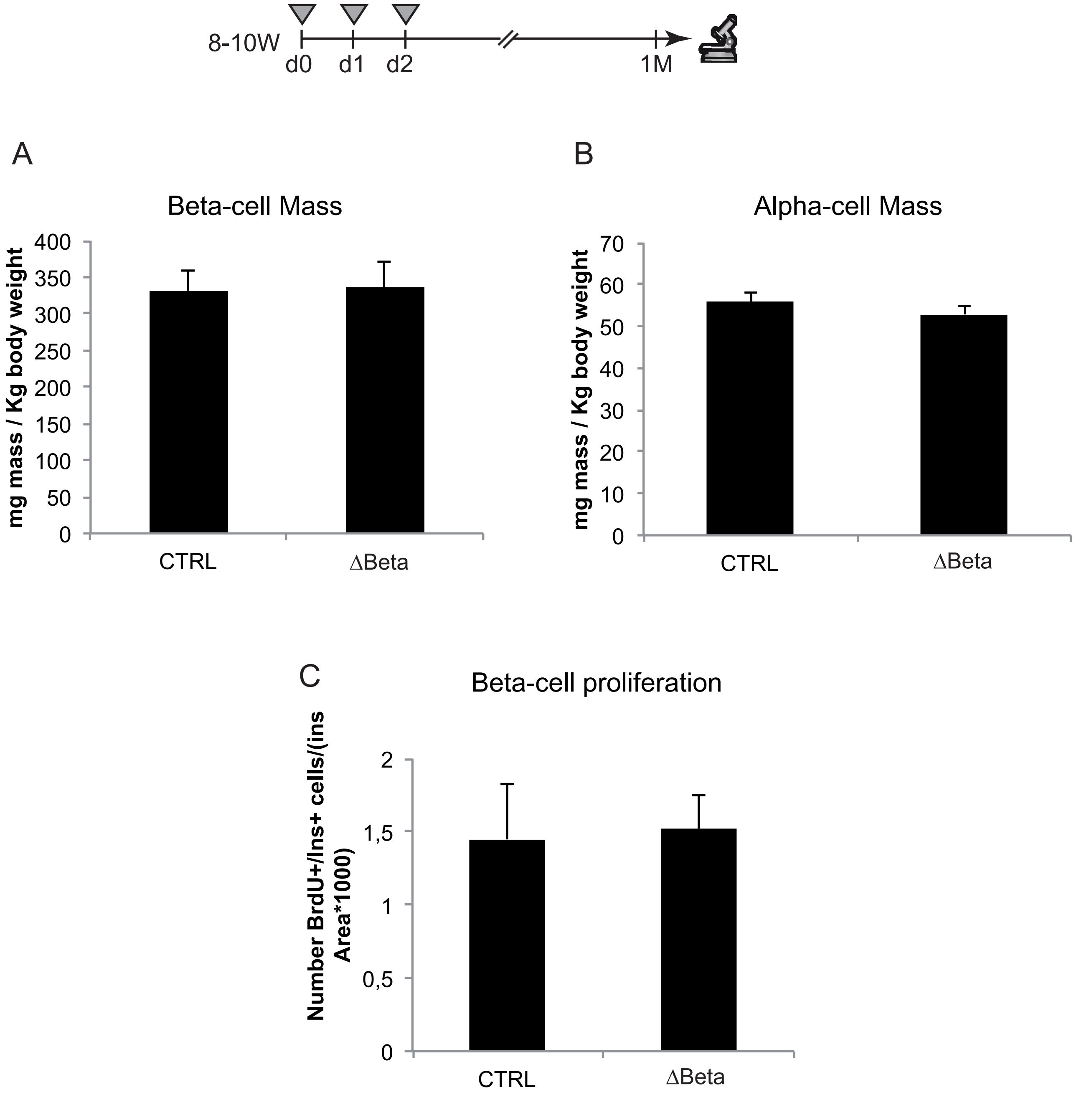

Figure S3: Data showing that alpha and beta cell mass as well as beta cell proliferation are not altered in adult Rfx6 ΔBeta mice one month after deletion of Rfx6 in beta cells, related to Figure 3.
Beta-cell mass (A) and alpha-cell mass (B) were quantified 1 month (M) after tamoxifen injections on n=4 control and Rfx6ΔBeta adult mice from insulin and glucagon immunofluorescence area and pancreas weight. Beta cell mass = (Ins Area Fraction/100)*PancreasWeight(g)*1000)/(MouseWeight(g)/1000). Ins Area Fraction = (Ins Area) *100 / (Dapi Area). Alpha cell mass was calculated the same way except that Gcg area was taken into account instead of Ins area. C) Controls and Rfx6ΔBeta mice were injected with BrdU 24h, before analyses and the number of Ins-positive/BrdU-positive cells was counted on n=4 animals 1 month after tamoxifen injections and the results normalized relative to the total Insulin area. Grey triangles indicate the days of tamoxifen injections.

	Gene name	Average expresion CTRL	Average Expression Rfx6Δbeta	FC	p-value
Rfx family members	Rfx1	60.45	44.22	-1.35	ns
	Rfx2	52.94	38.34	-1.35	ns
	Rfx3	1333.66	953.66	-1.37	ns
	Rfx4	1.20	1.77	1.28	ns
	Rfx5	659.73	454.97	-1.44	1.48E-03
	Rfx6*	1533.27	675.14	-2.23	2.40E-13
	Rfx7	130.23	118.77	-1.09	ns
	Rfx8	9.89	8.11	-1.18	ns
Beta cell function/transcription	Ins1	937814.39	498990.79	-1.85	2.74E-07
	Ins2	1914938.84	1896566.74	-1.01	ns
	Slc2a2	13015.56	9598.95	-1.34	4.43E-02
	Gck	2130.97	1344.81	-1.57	8.11E-05
	Abcc8	6540.14	4579.93	-1.41	1.31E-02
	Kcnj11	1197.27	1056.54	-1.13	ns
	Pcsk1	6639.56	7178.24	1.08	ns
	Pcsk2	26677.09	24193.81	-1.10	ns
	Slc30a8	19055.92	14614.63	-1.30	8.90E-03
	Chga	149119.97	129278.82	-1.15	ns
	Iapp	766631.71	678124.88	-1.13	ns
	Syp	3647.62	4329.68	1.18	ns
	Sytl4	6151.99	5810.84	-1.06	ns
	Ucn3	3889.29	1769.32	-2.12	2.45E-06
	Neurod1	1764.47	971.99	-1.80	7.27E-09
	Pdx1	840.71	623.79	-1.33	ns
	Nkx6-1	1805.28	1630.66	-1.10	ns
	Isl1	3906.73	2125.09	-1.81	1.54E-06
	Pax6	6600.11	3210.75	-2.03	7.73E-14
	Insm1	1043.29	856.28	-1.21	ns
	Mafa	2701.13	1948.29	-1.33	ns
	Mafb	271.77	202.74	-1.33	ns
	Mnx1	225.32	134.05	-1.66	2.01E-05
	Myt1	1994.96	1037.75	-1.87	8.57E-05
	Neurog3	41.81	29.20	-1.33	ns
	Nkx2-2	1110.44	878.78	-1.23	ns
	Sox4	2551.92	2895.45	1.12	ns
	Foxa2	1550.68	1341.41	-1.15	ns

Table S1 : Expression of selected genes in Rfx6 $^{\Delta beta}$ islets related to Figure 2 and 4
RNA Seq data on control and Rfx6 $^{\Delta beta}$ mouse islets (n=3), 5 days after Tamoxifen treatment. FC: mutant /controls (CTRL); ns: non significative p value > 0.05; Average expression: normalized read counts divided by gene length. * Only exon-3 of *Rfx6* is deleted in Rfx6$^{\Delta beta}$ mouse islets. Remaining exons (Rfx6 has 19 exons) can be transcribed and thus identified as “Rfx6” mRNA in the RNA sequencing experiments.

Gene name	Description	Fold Change Mutant/ctrl	p-value	Average expression (controls)	Average expression (mutants)
**Cacna1e**	calcium channel, voltage-dependent, R type, alpha 1E subunit	-4.90	4,89E-09	46.15	7.09
**Cacnb2**	calcium channel, voltage-dependent, beta 2 subunit	-2.10	3,79E-08	876.24	407.18
**Cacna1c**	calcium channel, voltage-dependent, L type, alpha 1C subunit	-2.17	1,54E-05	337.79	148.65
**Cacna1b**	calcium channel, voltage-dependent, N type, alpha 1B subunit	-1.82	6,28E-04	101.02	53.66
**Cacna1a**	calcium channel, voltage-dependent, P/Q type, alpha 1A subunit	-1.82	1,87E-03	2153.91	1129.47
**Cacna2d3**	calcium channel, voltage-dependent, alpha2/delta subunit 3	-2.10	2,78E-03	64.74	27.72
**Cacna1d**	calcium channel, voltage-dependent, L type, alpha 1D subunit	-1.66	3,52E-03	490.83	287.35
**Cacnb1**	calcium channel, voltage-dependent, beta 1 subunit	-1.54	7,14E-03	184.25	116.99
**Cacnb3**	calcium channel, voltage-dependent, beta 3 subunit	-1.15	ns	445.49	387.15
**Cacna2d1**	calcium channel, voltage-dependent, alpha2/delta subunit 1	-1.17	ns	1232.37	1051.85
**Cacng8**	calcium channel, voltage-dependent, gamma subunit 8	1.51	ns	0.88	4.36
**Cacna1s**	calcium channel, voltage-dependent, L type, alpha 1S subunit	1.48	ns	0.64	1.23
**Cacna1h**	calcium channel, voltage-dependent, T type, alpha 1H subunit	-1.14	ns	205.02	178.96
**Cacng1**	calcium channel, voltage-dependent, gamma subunit 1	1.24	ns	0.42	1.51
**Cacna1i**	calcium channel, voltage-dependent, alpha 1I subunit	-1.24	ns	102.85	77.25
**Cacng2**	calcium channel, voltage-dependent, gamma subunit 2	-1.25	ns	3.05	2.24
**Cacnb4**	calcium channel, voltage-dependent, beta 4 subunit	1.23	ns	1.35	1.73
**Cacng4**	calcium channel, voltage-dependent, gamma subunit 4	-1.20	ns	36.70	29.48
**Cacna2d2**	calcium channel, voltage-dependent, alpha 2/delta subunit 2	-1.18	ns	17.75	14.30
**Cacna1g**	calcium channel, voltage-dependent, T type, alpha 1G subunit	-1.09	ns	6.20	5.57
**Cacng7**	calcium channel, voltage-dependent, gamma subunit 7	1.13	ns	6.11	6.94
**Cacna2d4**	calcium channel, voltage-dependent, alpha 2/delta subunit 4	1.06	ns	5.23	5.67
**Cacna1f**	calcium channel, voltage-dependent, alpha 1F subunit	-1.05	ns	9.07	8.54
**Cacng5**	calcium channel, voltage-dependent, gamma subunit 5	-1.01	ns	1.88	1.84
**Cacng3**	calcium channel, voltage-dependent, gamma subunit 3	1.01	ns	1.92	1.90

Table S2: Down-regulation of voltage-dependent calcium channel (VDCC) genes in beta cells lacking Rfx6, related to Figure 5.
Table showing the expression (RNASeq) of voltage-dependent calcium channel genes in control (ctrl) and Rfx6 Δbeta mouse islets 5 days after Tamoxifen treatment. FC: mutant /control, ns: non specific $p<0.05$. Average expression: normalized read counts divided by gene length.

Gene name	Description	Fold Change	Average expression controls	Average expression RfX6 ∆beta
**Myl9**	myosin, light polypeptide 9, regulatory	5.73	311.40	2431.82
**Ogn #**	Osteoglycin	5.54	16.63	148.53
**Ly6c2**	lymphocyte antigen 6 complex, locus C2	5.43	2.56	69.89
**Islr #**	immunoglobulin superfamily containing leucine-rich repeat	5.36	5.53	45.81
**Tgm2**	transglutaminase 2, C polypeptide	5.18	254.50	1972.31
**Pdgfra (1)**	platelet derived growth factor receptor, alpha polypeptide	5.13	20.74	234.54
**Gas1 #**	growth arrest specific 1	4.76	14.30	151.42
**Gda**	guanine deaminase	4.72	91.06	873.70
**Pcolce #**	procollagen C-endopeptidase enhancer protein	4.47	69.00	445.47
**Igfbp4 (1) #**	insulin-like growth factor binding protein 4	4.15	373.64	2227.03
**Mylk #**	myosin, light polypeptide kinase	3.99	65.44	308.06
**Cd302 (1)**	CD302 antigen	3.85	28.43	161.11
**Mgst1 #**	microsomal glutathione S-transferase 1	3.85	125.96	748.69
**Cxcl12 (1)**	chemokine (C-X-C motif) ligand 12	3.76	49.09	229.09
**Arhgdib #**	Rho, GDP dissociation inhibitor (GDI) beta	3.70	51.28	309.85
**Itih5**	inter-alpha (globulin) inhibitor H5	3.58	14.48	73.07
**Tst #**	thiosulfate sulfurtransferase, mitochondrial	3.56	42.63	184.24
**Slc16a1 (1) #**	solute carrier family 16 (monocarboxylic acid transporters), member 1	3.51	16.55	85.38
**Fxyd1 #**	FXYD domain-containing ion transport regulator 1	3.39	35.07	152.43
**Ldha (1) #**	lactate dehydrogenase A	3.11	657.33	2693.39
**Fcgrt #**	Fc receptor, IgG, alpha chain transporter	3.11	76.96	333.51
**Maf #**	avian musculoaponeurotic fibrosarcoma (v-maf) AS42 oncogene homolog	3.06	15.36	79.90
**Sult1a1**	sulfotransferase family 1A, phenol-preferring, member 1	3.02	83.74	356.79
**Ddah1 #**	dimethylarginine dimethylaminohydrolase 1	2.94	6.70	24.03
**Gas6 #**	growth arrest specific 6	2.91	228.93	793.31
**Selenbp1**	selenium binding protein 1	2.80	35.91	129.27
**Car2 #**	carbonic anhydrase 2	2.64	11.34	43.97
**Gucy1a3 #**	guanylate cyclase 1, soluble, alpha 3	2.51	55.33	149.02
**Cat #**	catalase	2.50	153.94	428.03
**Ndrg2**	N-myc downstream regulated gene 2	2.48	45.05	121.75
**Nfib #**	nuclear factor I/B	2.45	133.27	345.35
**Yap1**	yes-associated protein 1	2.43	124.39	348.35
**Lmo4 #**	LIM domain only 4	2.40	59.70	161.98
**Tns1 #**	tensin 1	2.31	71.00	168.93
**Ly6a**	lymphocyte antigen 6 complex, locus A	2.23	332.03	833.66
**Smad3 #**	MAD homolog 3 (Drosophila)	2.22	346.47	795.26
**Hbb-b1**	hemoglobin, beta adult major chain	2.21	426.06	1331.47
**Hbb-b2**	hemoglobin, beta adult minor chain	2.20	73.60	234.06
**Parp3**	poly (ADP-ribose) polymerase family, member 3	2.15	47.28	115.77
**Fgf1 #**	fibroblast growth factor 1	2.12	601.17	1325.74
**Acot7 #**	acyl-CoA thioesterase 7	2.03	73.13	161.04
**Zfp36l1 #**	zinc finger protein 36, C3H type-like 1	2.03	41.87	91.95
**Oat (1) #**	ornithine aminotransferase	2.03	256.77	576.58
**Galm #**	galactose mutarotase	1.94	19.39	39.90
**Ly6c1**	lymphocyte antigen 6 complex, locus C1	1.81	326.64	626.69
**Ak3**	adenylate kinase 3	1.78	635.52	1161.10

**Mgll #**	monoglyceride lipase	1.70	462.81	831.93
**Bloc1s1 #**	biogenesis of lysosome-related organelles complex-1, subunit 1	1.70	121.03	215.11
**Nola2 (Nhp2) #**	NHP2 ribonucleoprotein homolog (yeast)	1.64	175.93	299.91
**Plec #**	plectin	1.50	190.71	286.16
**Zyx**	zyxin	1.40	57.71	82.45
**C1qbp**	complement component 1, q subcomponent binding protein	1.43	282.95	406.83
**Uqcrb**	ubiquinol-cytochrome c reductase binding protein	1.34	1598.62	2152.23
**Ndrg4**	N-myc downstream regulated gene 4	1.22	761.90	935.42
**Cox5a**	cytochrome c oxidase, subunit Va	ns	748.60	1008.20
**Crlz1 (Utp3) #**	small subunit processome component, homolog (S. cerevisiae)	ns	365.34	407.60
**Higd1a #**	HIG1 domain family, member 1A	ns	110.53	126.48
**Rpl24 #**	ribosomal protein L24	ns	3565.55	3452.93
**Rpl36 #**	ribosomal protein L36	ns	151.04	144.09
**Hba-a1**	hemoglobin alpha, adult chain 1	ns	94.77	207.23
**Hba-a2 #**	hemoglobin alpha, adult chain 2	ns	18.56	87.10
**Abca8a**	ATP-binding cassette, sub-family A (ABC1), member 8a	ns	193.17	296.45
**Mgst3 #**	microsomal glutathione S-transferase 3	ns	215.97	306.02
**Gsta4 #**	glutathione S-transferase, alpha 4	ns	65.32	104.21
**Fam59a #**	family with sequence similarity 59, member A	ns	15.55	19.85
**Aspa**	Aspartoacylase	ns	33.72	30.19
**Zdhhc9**	zinc finger, DHHC domain containing 9	ns	45.10	51.59

Table S3: Rfx6 represses disallowed genes (data related to Figure 6)
Disallowed genes as defined by Pullen et al Islet 2010 and Thorrez et al Genome Research 2010 are up-regulated when Rfx6 is deleted in adult beta-cells (RNA Seq data on control and Δbeta mouse islets 5 days after Tamoxifen treatment. FC: mutant/controls, ns, non significative p value > 0.05. Average expression: normalized read counts divided by gene length. (1) disallowed genes identified in both studies. # potential direct target of Rfx6 based on the identification of a peak in the vicinity of these genes in Rfx6 ChIPSeq experiments performed in Min6B1 cells (see Methods).
